# Transcriptomic Responses of MRSA to Penicillin-Modulating Phlorotannins Derived from *Pelvetia canaliculata*

**DOI:** 10.3390/md24080280

**Published:** 2026-08-12

**Authors:** James Blee, Peter O’Hara, Jialun Wu, Conor O’Byrne, Thomas J. P. Smyth, Owen Kenny

**Affiliations:** 1 Department of Health and Nutritional Science, Atlantic Technological University, F91 YW50 Sligo, Irelandpeter.ohara@research.atu.ie (P.O.);; 2 Bacterial Stress Response Group, University of Galway, H91 TK33 Galway, Ireland; 3 School of Life Sciences, Atlantic Technological University, F91 YW50 Sligo, Ireland

**Keywords:** antimicrobial resistance, antimicrobial, antibiotic modulation, biofilms, macroalgae, *Pelvetia canaliculata*, penicillins, RNAseq, transcriptomics

## Abstract

Methicillin-resistant *Staphylococcus aureus* (MRSA) infections contribute significantly to the mortality rate associated with antimicrobial resistance, while also increasing medical complications when compared to methicillin-sensitive isolates. Consequently, increasing the efficacy of β-lactams may alleviate the burden associated with this drug–pathogen combination. Dialysed extracts, derived from *Pelvetia canaliculata*, were further partitioned to generate phlorotannin enriched extracts (PEEs). Minimum inhibitory concentrations (MICs) of extracts were used to determine the antimicrobial and antibiotic-modulating activity against seven clinical MRSA isolates. Whole-transcriptomic sequencing was carried out to determine potential mechanisms of action on the strongest extract–antibiotic combination against an MRSA isolate, determined by the fractional inhibitory concentration index (FICI). The most pronounced β-lactam modulatory effects were seen in the >100 kDa PEE from *P. canaliculata* in conjunction with amoxicillin (MIC fold-reductions = 12.8–214), while >30 kDa PEE resulted in the strongest FIC index value when combined with penicillin (0.23), indicating a synergistic effect. Whole-transcriptome analysis of this treatment identified multiple differentially expressed genes (Log2FC +1/−1) of statistical significance (*p* adj < 0.05), namely related to accelerated autolysis, cell-envelope stability, protein synthesis, DNA replication, iron homeostasis and active transport. This study highlights preliminary transcriptomic evidence of biological processes associated with penicillin modulation by *P. canaliculata* > 30 kDa PEE, providing a foundation for further investigation of its antibiotic-modulating activity.

## 1. Introduction

The acceleration of antimicrobial resistance (AMR) in the absence of novel therapeutics is a major threat to global medicine. Globally, AMR was associated with 4.95 million deaths in 2019, of which 1.27 million were directly attributable to bacterial resistance [1]. Among the major contributors, methicillin-resistant *Staphylococcus aureus* (MRSA) accounted for the highest number of deaths (100,000) related to a single drug–pathogen combination [1]. Consequently, MRSA remains a major cause of both healthcare and community-associated infections [2].

The persistence of MRSA reflects a wider issue surrounding antibiotic usage and the growing selective pressure favouring resistant pathogens. Highlighting this is Europe’s 1% increase in antibiotic use between 2019 and 2023, with penicillins remaining the most frequently prescribed class in both community (47%) and hospital (34%) settings [3]. Clinically, MRSA infections are linked to prolonged hospital stays, increased rates of readmission, and elevated healthcare costs [4], while also incurring the second-highest number of disability-adjusted life years (DALYs) at 32.6 per 100,000 people [5]. Furthermore, projections estimate that AMR could result in 10 million deaths annually by 2050 if no effective interventions are implemented [6].

MRSA demonstrates multiple resistance mechanisms against β-lactam antibiotics, most notably (i) enzymatic degradation, (ii) biofilm formation, and (iii) the production of penicillin-binding protein 2a (PBP2a), which exhibits a low affinity for β-lactams [7]. Hospital-acquired MRSA (HA-MRSA) isolates generally display broader antimicrobial resistance profiles and are more frequently associated with bloodstream, urinary, and respiratory tract infections. In contrast, community-acquired MRSA (CA-MRSA) strains are commonly linked to skin infections such as impetigo and tend to exhibit greater intrinsic virulence [8]. A key distinction between these groups lies in the structure of their *Staphylococcal cassette chromosome mec* (SCC*mec*) elements. HA-MRSA typically carries the larger SCC*mec* types I–III, whereas CA-MRSA predominantly harbours the smaller SCC*mec* types IV and V. The reduced size of SCC*mec* IV and V is thought to facilitate greater genomic mobility in CA-MRSA, while also correlating with increased susceptibility to many non-β-lactam antimicrobial agents [9].

Among resistance mechanisms, biofilm formation is a major contributor to HA infections, as bacteria embedded within biofilms exhibit altered gene and protein expression relative to planktonic cells, resulting in enhanced persistence and antibiotic tolerance [10]. Biofilm-associated infections are linked to prolonged hospitalisation, increased morbidity and mortality, and elevated healthcare costs, and are estimated to account for more than 65% of nosocomial infections and 80% of chronic infections [11]. Bacteria within biofilms may exhibit 10–1000-fold greater antibiotic tolerance than their planktonic counterparts [12]. In particular, *S. aureus* biofilms have demonstrated 2–512-fold increases in minimum bactericidal concentrations (MBCs) for β-lactam/β-lactamase inhibitor combinations such as ampicillin–sulbactam and amoxicillin–clavulanate [13]. Thus, the identification of antimicrobial compounds that suppress biofilm development and retain bacterial cells in a planktonic state may increase the susceptibility of bacteria to antibiotic treatment, thereby enhancing antibiotic activity to isolates that already demonstrate significant resistance.

Compared to methicillin-sensitive *S. aureus* (MSSA), MRSA infections are often associated with higher antibiotic exposure, medical complications, and mortality rates, especially when biofilm formation is involved [14,15,16,17]. Over 70% of AMR-attributable deaths are linked to resistance against β-lactams and fluoroquinolones [1], underscoring the urgent need for the development of alternative therapeutics. Despite these trends, 47% of traditional antibiotics in the World Health Organisation (WHO) 2024 clinical pipeline remain focused on β-lactams or their resistance modulators, namely β-lactamase inhibitor combinations [18]. The prioritisation of β-lactams not only highlights the reliance of these antibiotics for treatment options but also suggests a stagnation in novel antibiotic development.

To address this challenge, the development of antimicrobials or antibiotic modulators with alternative pharmacophores to current antibiotics on the market is critical. As of June 2021, 25% of compounds currently in Phase-I of clinical development contain novel pharmacophores, while only 5% have novel mechanisms of action [19]. Marine macroalgae, particularly species within the *Phaeophyceae* phylum, represent promising sources of chemically diverse bioactive compounds known as phlorotannins. These are diverse hydrophilic polymers of phloroglucinol (1,3,5-trihydroxybenzene) that are structurally analogous to terrestrial hydrolysable tannins. Their diversity stems from their reported variations in degree of polymerisation (126 Da to 650 kDa), isomerisation, and bonding patterns, which allows classification into subclasses such as fucols, fucophlorethols, phlorethols/fuhalols, and carmalols [20].

Several studies have demonstrated the antimicrobial activity of phlorotannin-enriched extracts (PEEs) from the Fucales family. For instance, dieckol, a phlorotannin isolated from *Ecklonia stolonifera*, has shown potent anti-MRSA activity (MIC = 32–64 μg/mL) and synergistic effects with β-lactam antibiotics, reducing the required concentrations of ampicillin and penicillin (FIC = 0.06 and 0.26, respectively) [21]. Similarly, PEEs derived from *P. canaliculata* have demonstrated antimicrobial activity against both MSSA (MIC = 31.25–250 μg/mL) and MRSA (MIC = 125–500 μg/mL) [22]. While the antimicrobial and antibiotic-modulating activities of PEEs have been demonstrated previously, the molecular mechanisms responsible for these effects remain poorly understood. No study has comprehensively characterised the global transcriptomic response of MRSA following exposure to a PEE to identify the pathways associated with enhanced β-lactam susceptibility.

Therefore, this study first evaluated the antimicrobial and antibiotic-modulating activities of molecular weight-fractionated PEEs derived from *P. canaliculata* to identify the most biologically active fraction, before employing whole-transcriptome sequencing to investigate the molecular mechanisms underpinning these effects. By linking phenotypic activity with genome-wide transcriptional changes, this study provides new mechanistic insights into the pathways associated with β-lactam modulation in MRSA.

## 2. Results

### 2.1. Antimicrobial Activity

Fourteen extracts generated from seven *P. canaliculata* isolates were evaluated for their antimicrobial activity against four HA- and three CA-MRSA isolates (Table 1). Overall, the strongest antimicrobial activity was observed in the 30–100 kDa crude extract (CE) (MIC = 187.5–375 µg/mL), which demonstrated improved activity when compared to the initial crude aqueous extract (MICs = 750–2000 µg/mL).

All PEE fractions typically maintained or improved antimicrobial activity, while fucoidan-enriched extract (FEE) fractions exhibited a reduction in antimicrobial activity relative to their respective crude extract. A significant correlation (r = −0.645; *p* < 0.001) was found between total phloroglucinol concentration in all extracts and antimicrobial activity, while no correlation (r = 0.1339; *p* = 0.189) was found between total fucoidan content and antimicrobial activity, suggesting that phlorotannins are likely responsible for the observed antimicrobial effect. No antimicrobial activity was detected in the lower-molecular-weight fractions (MWCO < 3, 3–10, 10–30 kDa) at the tested concentration of both macroalgae, most likely due to the lower phloroglucinol content (Table S1).

### 2.2. Antibiotic Modulation

All extracts generated from *P. canaliculata* were assessed for their ability to modulate the activity of four β-lactam antibiotics (ampicillin, amoxicillin, oxacillin, and penicillin) against the seven MRSA clinical isolates previously evaluated for antimicrobial activity (Table 2 and Table 3). Modulatory activity was classified based on the FIC, where FIC ≤ 0.5 indicates synergism, and 0.5–1.0 indicates additive effects. In general, FIC values decreased with an increasing molecular weight of the extracts, while PEEs often exhibited enhanced modulatory effects compared to their corresponding crude extracts. For most fractions, modulatory activity was either improved or matched when compared to the crude extract at a reduced concentration relative to the MIC. The most pronounced modulatory effects were observed in the >100 kDa PEE in conjunction with amoxicillin across all MRSA isolates (MIC fold-reductions of 12.8–214), while >30 kDa PEE from *P. canaliculata* (1/8 MIC) resulted in the strongest FIC value (FIC = 0.23), resulting in a 10-fold-reduction in penicillin MIC against the MRSA HA 549 isolate. No β-lactam modulatory activity was detected in the lower-molecular-weight fractions of either species (<3, 3–10, and 10–30 kDa).

### 2.3. Time-Kill Kinetics of PEE Extracts in Combination with Penicillin

**Figure 1:**
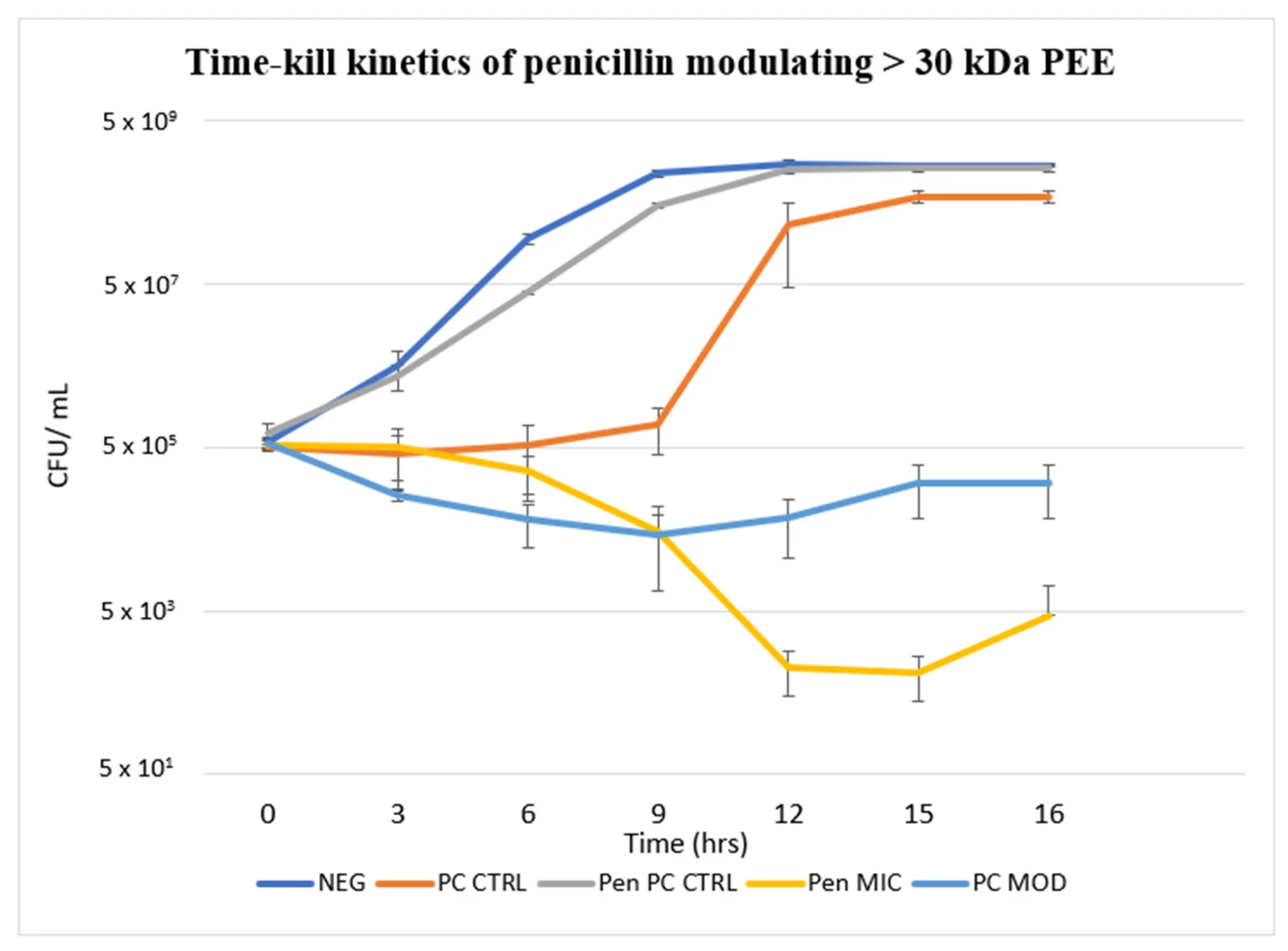
Time-kill kinetics of >30 kDa PEE from *P. canaliculata* (1/4 MIC) in combination with penicillin (PC MOD) at the modulating MIC against MRSA HA 549 isolate (*n* = 3). ‘PC CTRL’, the *P. canaliculata* extract control; ‘Pen PC CTRL’, penicillin MIC under modulation tested as a sole agent; ‘Pen MIC’, penicillin MIC (unchanged); ‘NEG’, negative control.

The time-kill kinetics of *P. canaliculata* > 30 kDa PEE 1/4 MIC modulatory activity in combination with penicillin were assessed against the HA-549 isolate. As can be seen in Figure 1, when either PEE or the penicillin combination was used alone, no effective inhibition was observed. However, upon respective combination, a bacteriostatic effect was observed (<3 log CFU/mL reduction).

### 2.4. Biofilm Inhibition

**Figure 2:**
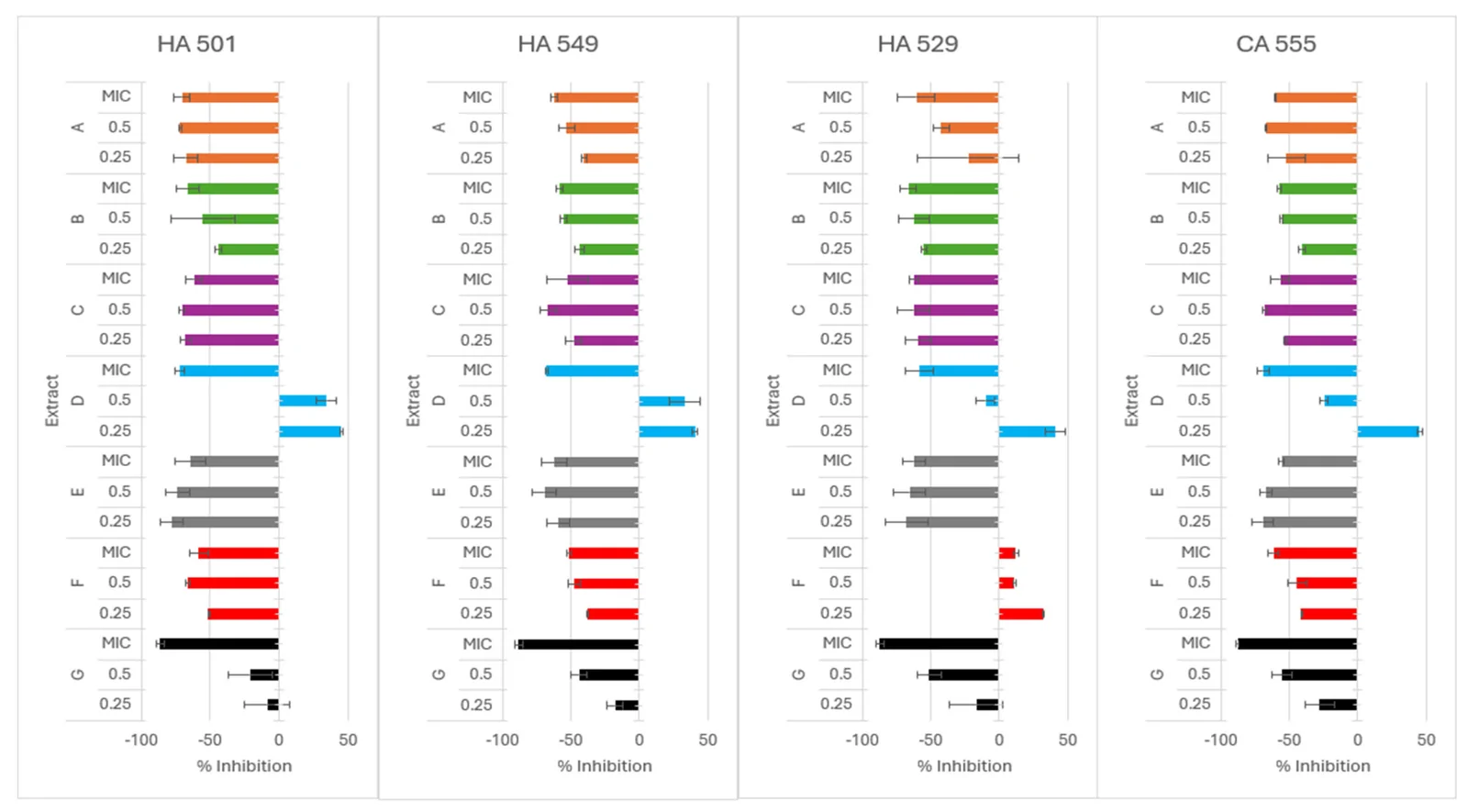
Biofilm inhibition (24 h) of *P. canaliculata* MWCO extracts (kDa), where A ≥ 30 CE; B ≥ 30 PEE; C = 30–100 CE; D = 30–100 PEE; E ≥ 100 CE; F ≥ 100 PEE; G = penicillin against three HA- and one CA-MRSA isolates, where error bars indicate standard deviation (*n* = 3). All extracts demonstrated biofilm inhibition, with the exception of extracts ‘D’ and ‘F’, which enhanced biofilm formation at subtherapeutic concentrations.

All crude and PEE extracts derived from *P. canaliculata* were assessed for biofilm inhibitory activity against three HA-MRSA and one CA-MRSA isolates (Figure 2). Overall, *P. canaliculata* extracts demonstrated universal activity across all isolates, except for 30–100 kDa PEEs, which increased biofilm production at subtherapeutic concentrations (1/2 and 1/4 MIC).

### 2.5. Whole-Transcriptomic Analysis

**Figure 3:**
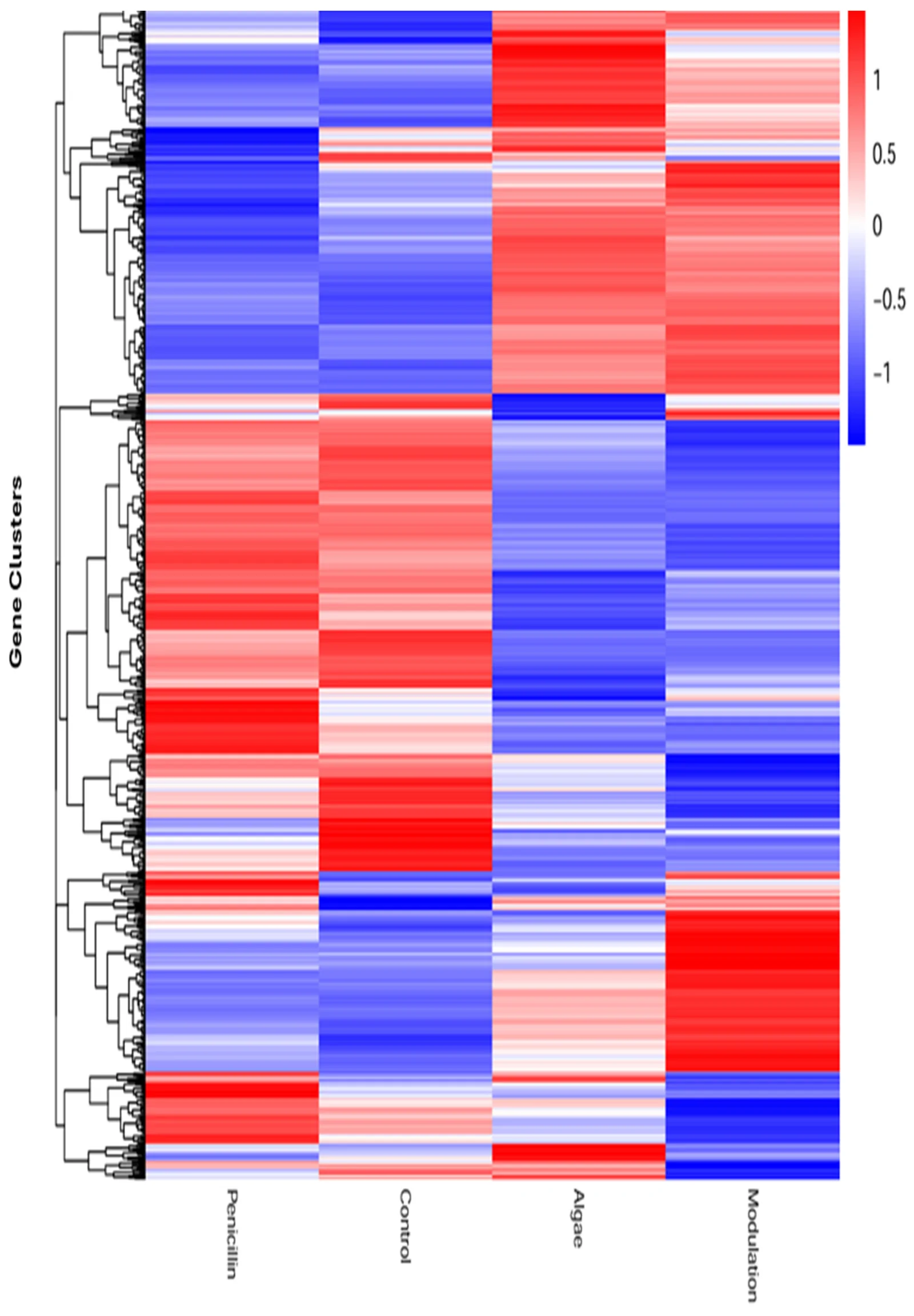
Heatmap cluster displaying differentially expressed genes (DEGs), where blue indicates downregulation and red indicates upregulation.

Whole-transcriptome analysis was employed to investigate the transcriptional changes associated with β-lactam modulation and the observed synergistic activity, with the aim of identifying stress-response pathways and candidate genes associated with enhanced susceptibility. To investigate this, the transcriptomic profile of MRSA HA 549 was analysed following treatment of subtherapeutic concentration (1/12 MIC) of *P. canaliculata* > 30 kDa PEE in combination with penicillin using the new MIC under modulation (0.47 µg/mL). A subtherapeutic concentration of extract was applied because (1) the optimal modulating treatment presents severe stress to bacterial cells and arrests the transcriptional response, and (2) a high concentration of extract interferes with RNA extraction. The transcriptomic analysis revealed 423 upregulated and 335 downregulated differentially expressed genes (DEGs) (log2 fold change ≥ ±1, *p* adj < 0.05) compared to the control (Figure 3). Kyoto Encyclopaedia of Genes and Genomes (KEGG) enrichment analysis of these DEGs revealed significant (*p* adj < 0.05) enrichment of metabolic pathways associated with nitrogen and histidine metabolism, fatty acid degradation and nucleotide sugar biosynthesis.

Biological processes relating to the structural constituent of ribosome and molecule activity, transporter activity and organic acid binding were also upregulated (*p* adj < 0.05) through Gene Ontology (GO) enrichment analysis. The DEGs listed in Table 4 were uniquely detected in the modulation sample and were absent in both the extract and penicillin controls under the defined thresholds (log2 fold change ≥ ±1, adjusted *p*-value < 0.05), indicating a distinct transcriptional response associated with the modulation treatment. When compared to the controls, several other genes in the modulation complex were also differentially regulated at a significant level (*p* adj < 0.05) (Table S7), although their log2 fold changes did not meet the predefined threshold (±1). Among these were genes associated with stress-response pathways, indicating altered expression of stress-related genes in the modulation treatment.

### 2.6. Cell Viability Assay

**Figure 4:**
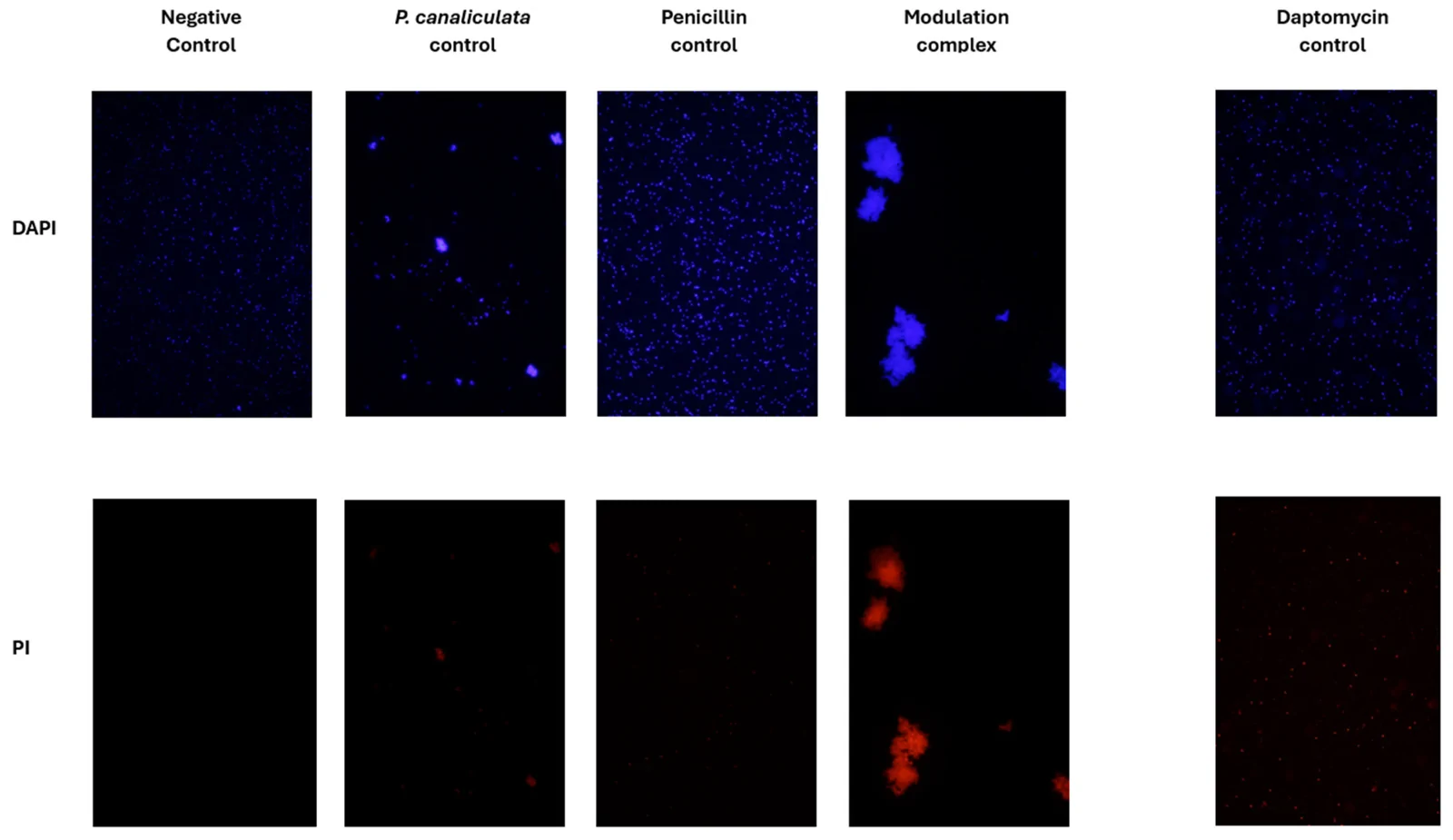
Cell viability assay following treatment of MRSA HA 549 isolate with *P. canaliculata* > 30 kDa PEE in combination with penicillin at the modulated concentration (*n* = 3). Blue regions (DAPI) indicate the presence of viable cells, while red regions indicate compromised cell membrane integrity (PI). Respective controls are listed, where *P. canaliculata* and penicillin used independently had minimal effect on cell damage. Daptomycin MIC was used as the positive control, while sterile water was used as the negative control.

*P. canaliculata* > 30 kDa PEE at a subinhibitory concentration (1/4 MIC) was assessed for its ability to alter cell membrane stability of the HA 549 MRSA isolate when combined with penicillin at its modulating concentration. As shown in Figure 4, treatment with either *P. canaliculata* > 30 kDa PEE (1/4 MIC) or penicillin alone produced only minimal effects on the isolate. In contrast, their combined application resulted in a marked reduction in cell membrane integrity, suggesting enhanced penicillin uptake due to increased membrane permeabilisation. Additionally, the combination treatment promoted noticeable bacterial cell aggregation.

## 3. Discussion

The present study demonstrates the antimicrobial, antibiofilm, and β-lactam-modulating activities of *P. canaliculata* aqueous extracts and presents the first transcriptomic analysis of the MRSA response to a PEE derived from this species. While the β-lactam-modulating activity of *P. canaliculata* has remained largely unexplored, whole-transcriptome sequencing revealed genome-wide transcriptional changes associated with treatment with the >30 kDa PEE and penicillin, providing mechanistic insight into the biological processes and pathways that may contribute to the observed enhancement of β-lactam susceptibility. By integrating phenotypic findings with transcriptomic analysis, this study identifies transcriptional responses associated with β-lactam modulation in MRSA.

### 3.1. Antimicrobial Activity

The significant correlation (r = −0.645; *p* < 0.001) found between total phloroglucinol concentration and antimicrobial activity indicates the role of phlorotannins in relation to the observed activity. Similarly, a correlation between phlorotannin content and antimicrobial activity (r = 0.55) has previously been reported by Kopf et al. [23].

In the present study, 30–100 CE (MICs = 187.5–375 µg/mL) and >30 kDa PEE (MICs = 250–375 µg/mL) demonstrated the strongest antimicrobial activity. Comparable results have been reported by Meshalkina et al. [22], where the antimicrobial properties of PEEs derived from *P. canaliculata* were reported against one MRSA (MIC = 125–500 µg/mL) and two MSSA (MIC = 31.2–125 µg/mL) isolates. Notably, a previous study assessing phlorotannin diversity and the degree of polymerisation (DP) of these extracts using HPLC-ESI-MS found a diverse abundance of high-molecular-weight phlorotannins (DP = 17–33) [24].

The low phloroglucinol content of lower-molecular-weight fractions (<3 kDa, 3–10 kDa, 10–30 kDa) (Table S1) may have influenced the limited antimicrobial activity of these extracts, which may have contained a high relative concentration of mannitol. This sugar alcohol is found abundantly in Phaeophyceae [25] and is used as a nutritive source by MRSA isolates [26], which may have potentially aided their growth.

### 3.2. Antibiotic Modulation

The findings outlined in Table 2 and Table 3 are consistent with previous studies involving phlorotannin-rich extracts. However, a large proportion of these studies are associated with lower-molecular-weight phlorotannins with smaller DPs. For example, Lee et al. isolated phlorotannins such as fucofuroeckol-A (DP = 5) from *Eisenia bicyclis*-modulated streptomycin activity with comparable FIC values (0.188–0.563) and showed additive effects in combination with erythromycin and lincomycin against *Propionibacterium acnes* (FICI = 0.5–0.751) [27]. Phlorofucofuroeckol-A (DP = 5) from the same species exhibited synergy with ampicillin, penicillin, and oxacillin against an MRSA isolate (FICI = 0.393–0.422), which was later linked to downregulation of *mecI*, *mecR1*, and *mecA* gene expression and increased membrane permeability [28]. Similarly, dieckol (DP = 6), a phlorotannin isolated from *E. stolonifera*, demonstrated potent synergism with ampicillin and penicillin against several MRSA clinical isolates (FICI = 0.078–0.266) [21], in which the mechanism of action was hypothesised as damage to the cell wall. Notably, oxacillin consistently showed the weakest modulation (FICI = 0.156–1.25) [21], which is in agreement with the present study, possibly due to structural or mechanistic factors affecting interaction with β-lactam resistance pathways. In addition, synergistic activity of an ethyl acetate extract from *Ecklonia cava* was seen following the combination with streptomycin against four *Listeria monocytogenes* strains (FICI = 0.141–0.531), with phlorotannins hypothesised as the key active components [29].

Conversely, phlorotannins isolated from *Hizikia fusiforme* failed to enhance the efficacy of levofloxacin or amikacin against *Pseudomonas aeruginosa* [30]. This lack of activity is likely due to the intrinsic barrier imposed by the outer membrane of Gram-negative bacteria, which restricts the entry of larger molecules. It has been reported that compounds targeting Gram-negative pathogens must have an average molecular weight of approximately 414 Da or less to penetrate the outer membrane [31]. Therefore, the application of phlorotannins as antibiotic modulators may be more viable against Gram-positive bacteria, which lacks this permeability barrier.

### 3.3. Biofilm Inhibition

MRSA biofilm formation was inhibited by a wide range of crude and PEEs. However, subtherapeutic concentrations of 30–100 kDa PEE were shown to stimulate biofilm formation. A previous study by Liu et al. [32] reported similar effects, where an increase in biofilm formation of *S. aureus* was witnessed following the application of 1/2 and 1/4 MIC of tetracycline and streptomycin. It was found that numerous biofilm-associated genes were upregulated during subtherapeutic treatment. Moreover, it is suggested that this response is induced following a sublethal dosage that results in a minor population of dead bacterial cells, leading to the release of their organelles (e.g., eDNA, cell fragments) and consequently stimulating biofilm formation [33].

Despite the increased interest in the antimicrobial potential of macroalgae-derived compounds, there remains limited available information to explain the biofilm-inhibitory properties of PEEs. As noted by Besednova et al. [34], the ability of macroalgal extracts to inhibit biofilm formation remains relatively unreported, and to date, no investigations have reported biofilm inhibitory activity for PEEs derived from *P. canaliculata*. To the best of our knowledge, this is the first study to describe such effects. Phlorotannins derived from other macroalgal species, such as *Hizikia fusiforme*, have shown anti-quorum sensing activity by reducing the availability of signalling molecules for bacterial receptors, potentially leading to inhibition of biofilm formation [30]. The results of this study suggest that the biofilm inhibitory activity observed at the same sub-MIC concentrations (1/4 MIC) used for β-lactam modulation, particularly for *P. canaliculata*, may contribute to the extract’s modulatory mechanism.

### 3.4. Whole-Transcriptomic Analysis

#### 3.4.1. Decreased Virulence and Induced Autolysis

The regulation of numerous virulence factors in MRSA is mediated by global regulatory systems, notably the staphylococcal accessory regulator A (*sarA*) and the accessory gene regulator (*agr*) system [35]. These systems encode DNA-binding proteins that exert widespread effects on gene expression [36]. Notably, *sarA* and *agr* often display an inverse correlation in biofilm regulation; downregulation of *sarA* combined with upregulation of *agr* has been shown to inhibit biofilm formation [37]. However, *sarA* is generally regarded as the dominant regulator of biofilm development, functioning independently of the *agr* system.

In the present study, *sarA* was significantly downregulated within the modulation complex, suggesting that the >30 kDa PEE derived from *P. canaliculata* effectively inhibits biofilm formation and maintains cells in a planktonic state, consistent with the observations presented in Figure 2. This finding aligns with previous reports demonstrating that *sarA* mutants exhibit increased production and secretion of extracellular nucleases and proteases, which disrupt biofilm formation [38,39].

Beyond its role in biofilm regulation, *sarA* also represses the expression of murein hydrolases, enzymes responsible for peptidoglycan turnover during cell division. Given their potential to induce autolysis, the expression of these enzymes is tightly controlled. It has previously been demonstrated that *sarA* mutants of *S. aureus* display increased rates of autolysis when exposed to penicillin, attributable to altered regulation of murein hydrolase activity [40]. More recently, it has been reported that vancomycin resistance in *S. aureus* is modulated by *sarA* through suppression of autolysin expression [41].

Consistent with these findings, several studies have shown that downregulation of *sarA* enhances β-lactam susceptibility in MRSA. For example, synergistic treatment with cinnamaldehyde has been shown to inhibit biofilm formation while increasing β-lactam efficacy via *sarA* suppression against MRSA isolates (FIC = 0.25–0.5) [42]. Similarly, hypericin has demonstrated the ability to both inhibit biofilm formation and potentiate β-lactam antibiotics, such as oxacillin, through inhibition of *sarA* expression [43].

Collectively, these findings suggest that downregulation of *sarA* may be associated with the enhanced β-lactam activity observed with the *P. canaliculata* > 30 kDa PEE against the MRSA HA 549 isolate.

#### 3.4.2. Cell Envelope and Antibiotic Resistance

The significant upregulation of genes encoding proteins containing Pfam domains PF00905 and PF03717, associated with PBP transpeptidase activity and dimerisation, suggests activation of cell wall stress responses and peptidoglycan remodelling. Moreover, the differential expression of several genes associated with cell wall repair and resistance indicates structural damage to the peptidoglycan layer. Concurrent enrichment of KEGG pathways associated with nucleotide sugar biosynthesis and fatty acid degradation suggests a compensatory cellular response to replenish membrane and cell wall precursors.

The observed upregulation of genes such as *cwrA* and *yvcK* further supports a strong cell envelope stress response. In *S. aureus*, *cwrA* is known to be upregulated following β-lactam exposure, where it contributes to restoring cell wall integrity [44]. Similarly, *yvcK* plays a critical role in maintaining cell shape and peptidoglycan synthesis during cell wall stress [45,46]. These responses are consistent with increased permeability of the cell wall to β-lactam antibiotics.

Phlorotannins, like other tannins, are known to disrupt membranes by precipitating proteins through hydrogen bonding and hydroxyl group interactions [47]. This membrane-disruptive capability was supported through the cell viability assay (Figure 4), which demonstrated reduced membrane integrity following combination treatment, compared to either agent alone. This increased permeability is likely to have facilitated greater penicillin uptake, contributing to the observed synergistic antimicrobial activity. This is further supported by the upregulation of DEGs including *drp35* and *lrgB*, which were exclusively observed in the modulation complex and are reported to be induced in response to both β-lactam exposure and membrane damage [48,49]. In a previous study by Beltrame et al. [50], *lrgB* mutant strains of MRSA display increased programmed cell death. In contrast, the upregulation of *lrgB* in the present study suggests a stress response aimed at decreasing autolysis following increased cell wall damage. In agreement with these findings, *vraE* upregulation was observed in *S. aureus* following treatment with telavancin, and it was suggested that *vraE* expression is indicative of membrane depolarisation [51]. Moreover, in the present study, genes (*vraE* and *uhpT*) associated with resistance for cell wall-targeting antibiotics such as bacitracin and fosfomycin were upregulated, indicating a coping mechanism for additional cell wall stress [52,53].

Supporting this, low-molecular-weight phlorotannins from *Sargassum thunbergii* have previously been shown to damage both the cell wall and membrane of *Vibrio parahaemolyticus* [54]. In the present study, notable cell aggregation and enhanced cell-to-cell adherence were observed following the combination of *P. canaliculata* > 30 kDa PEE and penicillin (Figure 4), suggestive of altered surface charge properties. Notably, significant upregulation of *dltC* and *dltD* genes, which are involved in the D-alanylation of lipoteichoic acid of *S. aureus,* was observed, resulting in a reduction in cell surface negative charge and an increase in cell surface hydrophobicity. *S. aureus* isolates expressing *dltC* have been associated with increased resistance to hydrophilic antimicrobials [55], potentially as a mechanism to reduce phlorotannin and penicillin effects, while *dlt*-mediated aggregation has been reported in *Streptococcus* species [56].

#### 3.4.3. Protein Synthesis Disruption

Ribosomes are essential cellular components integral to protein synthesis, comprising the small 30S subunit and the larger 50S subunit, both of which are common antibiotic targets [57]. In this study, treatment with the *P. canaliculata*- penicillin modulation complex significantly reduced ribosomal functioning by impairing the assembly of the 30S ribosomal subunit.

The *rpsT* gene, which encodes the 30S ribosomal protein S20, was significantly downregulated following treatment. S20 is a key component in the early stages of 30S subunit assembly, stabilising the 16S rRNA and facilitating correct mRNA alignment during translation [58]. Downregulation of *rpsT* has been associated with reduced protein synthesis efficiency and cellular fitness [59], while its deletion leads to defective mRNA binding and elevated levels of mistranslated proteins [60]. Additional downregulation of *rpsP* and *rpsN*, both involved in 30S subunit assembly, reinforces the conclusion that small subunit synthesis was significantly impaired. While it has been noted that rpsN is essential for growth, its depletion results in the formation of defective 30S subunits, likely increasing translational stalling [61]. Notably, the rpsN protein is zinc-dependent [61], which, in the present study, genes encoding zinc uptake proteins, including those containing the Pfam domain PF10297, were correspondingly downregulated, potentially indicating reduced protein synthesis.

Furthermore, there was simultaneous upregulation of *ssrA,* a gene that codes for a tmRNA molecule. tmRNA is recruited upon an incomplete or untranslatable polypeptide, resulting in stalled ribosomes, which subsequently tags the corresponding polypeptide for degradation [62]. Downregulation of genes involved in unfolding and degrading damaged proteins such as *clpP* was also seen (*p* adj < 0.05). A previous study [63] found that *clpP* mutants demonstrated severely attenuated virulence and increased protein misfolding, while also reducing the transcription of RNA III, an important effector molecule for the agr quorum-sensing system. These results suggest that modulation-induced S20 deficiency may increase the accumulation of defective proteins and ribosome stalling, while downregulation of *clpP* indicates an impaired capacity for protein quality control, likely exacerbating intracellular stress. A potential reduction in translational accuracy is further suggested by the significant downregulation of *queH*, which encodes an enzyme involved in the final step of queuosine biosynthesis for tRNA modification, a process known to enhance translational fidelity [64].

In contrast, several genes encoding 50S ribosomal subunit components such as *rpmB, rplS,* and *rpmE* were significantly upregulated (*p* adj < 0.05). This may represent a compensatory mechanism aimed at maintaining translational function despite 30S subunit insufficiency. Similar regulatory patterns have been reported in response to other polyphenolic compounds. For example, tannins from *Phyllanthus columnaris* have been shown to downregulate genes encoding both 30S and 50S subunits [65], suggesting that ribosomal disruption is a shared mechanism among tannin-rich extracts. Furthermore, *catB* was significantly upregulated. This gene encodes chloramphenicol acetyltransferase, an enzyme that inactivates chloramphenicol by acetylation, thereby reducing its ability to target the ribosome in MRSA [66]. This upregulation suggests a compensatory resistance response to antimicrobial pressure acting on ribosomal functioning.

These findings suggest that the modulation treatment is associated with transcriptional changes in genes involved in protein synthesis, including 30S ribosomal subunit biogenesis, mRNA translation, and protein quality control.

#### 3.4.4. Inhibition of DNA Replication

DNA replication is an essential and tightly regulated process in *S. aureus*, driven by the coordinated activity of DNA polymerase III. Namely, *polC* encodes PolC (POL III C), the main DNA polymerase in *S. aureus.* PolC possesses intrinsic proofreading capability via its 3′–5′ exonuclease domain, enabling both DNA synthesis and mismatch repair [67,68]. In the present study, treatment with the modulation complex resulted in significant downregulation of *polC*, suggesting a direct impairment of DNA replication in MRSA. Furthermore, *norB*, which encodes a multidrug efflux pump associated with resistance to DNA-targeting antibiotics such as quinolones in MRSA [69], was significantly downregulated. This may indicate a reduced efflux capacity, potentially limiting the ability of the isolate to export phlorotannin compounds and thereby increasing intracellular compound retention.

The downregulation of *polC* was accompanied by decreased expression of *holB*, which encodes the delta subunit of DNA polymerase III. The repression of *holB* may contribute to reduced PolC function or expression, further compounding replication stress. In parallel, *rpmG* was also downregulated, which plays an important role in mismatch and repair, indicating a diminished capacity for correcting replication-associated errors. This may increase the accumulation of mutations and contribute to genomic instability under treatment conditions [70]. The observed repression of these replication-associated genes points toward a multifaceted inhibition of DNA synthesis and repair pathways. Inhibition of PolC has been an attractive target for novel antimicrobial development due to its highly conserved nature across Gram-positive pathogens such as *Staphylococcus*, *Streptococcus* and *Enterococcus* [71]. The current findings suggest that phlorotannin-based modulation may exploit this vulnerability, contributing to the modulatory effects observed.

#### 3.4.5. Iron Homeostasis and Metabolic Shift

Iron is a critical micronutrient for bacterial viability, serving as a cofactor in essential processes such as DNA synthesis, ATP generation, and enzymatic catalysis [72]. As such, identifying novel agents that trigger iron-restricted conditions during a pathogenic infection has been proposed as a potential strategy for antibiotic modulation therapy [73]. During infections within the human body, hepcidin, which regulates iron homeostasis, causes a reduction in plasma iron and increases macrophage sequestration of iron, resulting in an iron-depleted environment to limit the replication of pathogens [74].

The iron-chelating potential of tannins, including PEEs, has been previously proposed as a mechanism contributing to their antimicrobial activity [75,76]. Transcriptomic data from the present study indicates a marked disruption in iron homeostasis following treatment with the modulation complex. Significant downregulation of haem storage genes (*ftnA*) suggests a depletion of intracellular iron reserves, while the concurrent upregulation of the *fecCD* transporter complex reflects a compensatory response to increase ferric iron uptake. In iron-limited conditions, *S. aureus* is known to redirect metabolic flux away from iron-dependent pathways such as the tricarboxylic acid (TCA) cycle toward glycolytic pathways [77]. This metabolic shift was supported by the upregulation of *arcA*, a regulator that represses TCA cycle activity [78], along with genes such as *yvcK* and *sdaAA*, which promote glycolytic metabolism [45]. KEGG pathway analysis further revealed the suppression of genes involved in oxidative phosphorylation and ATP-binding cassette (ABC) transport systems, consistent with an energy metabolism shift under iron restriction.

Acidification resulting from increased glycolytic activity may promote iron release from host iron-sequestering proteins [79]. Accordingly, the observed upregulation of *ureC* and *arcD*, genes involved in the production of ammonia and other alkali metabolites, suggests a compensatory response to intracellular acid stress. *UreC* encodes urease, which hydrolyses urea to produce ammonia at a low pH [80]. Meanwhile, *arcD* facilitates arginine catabolism, contributing to ammonia generation and energy production [81]. In parallel, genes involved in histidine metabolism were significantly upregulated, further supporting the role of amino acid metabolism in maintaining pH homeostasis under stress [82]. Moreover, a previous study [83] found that upregulation of *dlt* genes in *Lactococcus lactis* F44, such as *dltD* and *dltC* in this study, increased acid tolerance by increasing the overall positive charge on cell membranes.

During periods of iron scarcity, virulence-associated genes are often upregulated by downregulating the iron-dependent repressor *fur* (ferric uptake regulator) [84], thereby upregulating iron acquisition systems. In this study, the upregulation of virulence factors, such as *hlgABC* (the alpha-hemolysin operon) and *sbnC*, was observed, with *sbnC* representing a key component of the staphyloferrin B siderophore biosynthetic pathway. These systems facilitate iron acquisition via haemolysis and iron chelation [85], underscoring the host-adaptive responses of *S. aureus* under phlorotannin-induced iron stress.

#### 3.4.6. Reduced Active Transport and Export

The ATB-binding cassette (ABC) transporter family are transmembrane proteins that facilitate the import of nutrients into the cell and the exportation of harmful substances out of the cell (e.g., xenobiotics) [86]. In this study, several genes (*ydbJ*, *yhaQ*) involved in the KEGG ABC transporter pathway were significantly downregulated, including ABC transporter protein domains such as PF00950, in addition to ABC-2 family transport protein (PF12730) responsible for drug efflux. The observed repression of these transporters suggests a diminished capacity for the active efflux of antimicrobial agents, such as phlorotannins, potentially resulting in elevated intracellular accumulation. Conversely, the upregulation of particular ABC transporters (Table S7) may reflect a stress adaptation response aimed at maintaining minimal transport functionality under adverse conditions. Nevertheless, targeting ABC transporters in MRSA may provide an alternative strategy to combat AMR [87].

Although phlorotannins have shown promising potential as antibiotic modulators, the molecular basis for their modulatory activity has remained poorly understood. Notably, this study represents the first comprehensive transcriptomic investigation of the MRSA response to a PEE derived from *P. canaliculata* in combination with penicillin. Whereas previous studies have primarily demonstrated the antimicrobial or antibiotic-modulating activity of PEEs, the present work links these phenotypic effects to genome-wide changes in gene expression, providing mechanistic insight into the cellular pathways associated with β-lactam modulation. These findings advance the current understanding of phlorotannin-mediated antibiotic modulation and provide a molecular framework for future studies investigating marine-derived antibiotic modulators.

## 4. Materials and Methods

### 4.1. Chemicals and Reagents

Organic solvents (methanol, sulfuric acid, ethanol, formic acid, acetic acid) were purchased from Lennox laboratory supplies (Dublin, Ireland). The β-lactam antibiotics (penicillin, oxacillin, daptomycin, ampicillin, and amoxicillin), nitrocefin discs, Muller–Hinton broth, nutrient agar, crystal violet, safranin, iodine, sodium carbonate (Na_2_CO_3_), Folin–Ciocalteu (2N) reagent, safranin, iodine, propidium iodide (PI), 4′,6-diamidino-2-phenylindole, (DAPI), phloroglucinol, fucose, molecular weight cut-off tubes (<3 kDa, >10 kDa, >30 kDa, >100 kDa), Invitrogen Turbo DNase, RNAlater and iodonitrotetrazolium chloride (INT) were purchased from Sigma Aldrich (Dublin, Ireland). A RNeasy Lysis Tissue (RLT) buffer Qiagen RNeasy Mini Kit was purchased from Qiagen (Hilden, Germany).

### 4.2. Bacterial Strains and Culture Conditions

*Staphylococcus aureus* (ATCC 25923) was purchased from the American Type Culture Collection (ATCC). Clinical HA- and CA-MRSA isolates were donated by Sligo University Hospital (SUH), Ireland and were positively typed for the *mecA* gene by SUH. All clinical isolates tested positive, while *S. aureus* tested negative for β-lactamase production following nitrocefin application, as according to the manufacturer’s guidelines (MERCK 49862). All strains were stored on ceramic beads in glycerol at −80 °C. Prior to use, beads were streaked onto fresh nutrient agar plates and grown for 24 h at 37 °C. A single colony was inoculated into 15 mL of Muller–Hinton broth and incubated for 16 h at 37 °C. The inoculum was adjusted to a final testing concentration of 5 × 10^5^ CFU/ mL prior to testing. The testing inoculum was confirmed via serial dilution and a plate count on nutrient agar, which was incubated at 37 °C for 24 h. A Gram stain was performed on the tested inoculum to ensure a pure culture.

### 4.3. Sample Collection

A dried sample of *P. canaliculata* raw biomass was purchased from SEALAC Ltd. (Mayo, Ireland). All samples were ground into a fine powder using a Waring Laboratory Science™ LB20EG blender (Faust Lab Science GmbH, Kamen, Germany) at 20,000× *g*, vacuum packed and stored at −18 °C until further use.

### 4.4. Sample Extraction and Molecular Weight Cut-Off (MWCO) Generation

Briefly, ultrapure water was added to a ground sample (10:1) under constant agitation (150 rpm) at room temperature. Water containing the extracted materials was collected by centrifugation at 4000× *g* for five minutes at room temperature after 3 h. The supernatants were pooled, and the insoluble pellet was subjected to exhaustive extraction under the same conditions outlined above, for additional 3 h and 18 h periods. Pooled samples were subjected to molecular weight cut-off (MWCO) using dialysis tubing of varying molecular weights (3, 10, 30, 100 kDa). Crude aqueous extracts were solubilised in water, sonicated (Elma S180 sonicator (37 kHz), Elma Ultrasonic, Germany) for 10 min and centrifuged at 4000× *g* for five minutes at room temperature. Following that, 15 mL of the supernatant was added to a respective MWCO tube, which underwent further centrifugation at 4000× *g* for 45 min at room temperature. Respective fractions were pooled and dried as previously described above and stored at −18 °C in amber vials until further use.

MWCO fractions demonstrating the strongest antimicrobial activity were subjected to further bioactivity-guided fractionation. As previously described by Zayed et al. [88], the aim was to partition two analytes commonly found in Phaeophyceae (phlorotannins and fucoidan) into respective fractions. Selected MWCO fractions (100 mg) were solubilised using 45 mL of 70% ethanol (*v*/*v*), sonicated (Elma S180 sonicator (37 kHz), Elma Ultrasonic, Germany) for 10 min and stored at 4 °C. Following a 3 h extraction, samples were centrifuged at 4000× *g* for five minutes at 4 °C. The supernatant was pooled, and the pellet was subjected to additional 3 h and 18 h extractions under the same outlined conditions. The pooled supernatant was deemed the PEE, while the insoluble pellet was labelled as the fucoidan-enriched extracts (FEEs). The 70% ethanol (*v*/*v*) was removed using a rotary evaporator (Buchi Rotavapor R-3000, Flawil, Switzerland) at 40 °C under vacuum. The remaining aqueous extract and FEEs were lyophilised as previously described and stored at −18 °C in amber vials until further use.

### 4.5. Antimicrobial Activity

The broth microdilution method was used to determine the antimicrobial activity of extracts, as previously described by Smyth et al. [89]. All extracts (4000 μg/mL) were prepared by solubilising using sterile deionised water. Extracts were sonicated (Elma S180 sonicator (37 kHz), Elma Ultrasonic, Singen, Germany) for 10 min and centrifuged at 4000 rpm for 5 min using a Sorvall ST4R plus centrifuge. From the 4000 μg/mL stock, a 3000 μg/mL working solution was also prepared.

Using a 96-well microtiter plate, the following controls were added to lane one: 100 µL of Muller–Hinton broth (blank control), 100 µL of ampicillin (500 μg/mL) positive control), 100 µL of bacteria isolate and 100 µL of water (negative control). In the remaining wells, a 2-fold serial dilution of each extract was performed across corresponding rows using Muller–Hinton broth. Briefly, 200 µL of the 4000 μg/mL extract was added to 3 rows of lane 1, while 200 µL of the 3000 μg/mL extract was added to 3 rows of lane 2 (3 technical replicates). Next, 100 µL of Muller–Hinton broth was added to testing lanes 3–12, where a 2-fold serial dilution of each extract was performed across corresponding rows. For example, 100 µL of the 4000 μg/mL extract in lane 1 was transferred to lane 3, inverted thrice and was subsequently transferred to lanes 5, 7, 9 and 11 using the same procedure. An identical procedure was carried out for the working solution of 3000 μg/mL to lanes 4, 6, 8, 10 and 12, respectively. The testing of two different concentrations allowed for the identification of a more refined MIC. The final extract concentrations in lanes 11 and 12 were 62.5 μg/mL and 46.8 μg/mL, respectively. A volume of 100 µL of bacterial culture (5 × 10^5^ CFU/ mL) was added to all corresponding wells excluding the blank control. Plates were incubated at 37 °C for 18 h. Following incubation, 40 µL of iodonitrotetrazolium dye (INT; 0.2 mg/mL) was added to each well and was further incubated for 1 h at 37 °C. The application of INT is a well-known indicator of microbial respiration, where its reduction and consequent colour change from clear to red indicate cell viability. The last well where no colour change was observed after incubation was determined as the MIC. Tests were conducted using 3 biological replicates.

### 4.6. Antibiotic Modulation

Extracts were evaluated for their ability to modulate the activity of four β-lactam antibiotics (ampicillin, amoxicillin, oxacillin, and penicillin) against the outlined MRSA isolates using the fractional inhibitory concentration index (FICI), as previously described by Embaby et al. [90]. Antibiotic MICs were determined as described in Section 4.5.

Briefly, 100 µL of antibiotic solution at either 1/2 MIC or an intermediate concentration was dispensed into the appropriate wells of a 96-well microtiter plate (three technical replicates per concentration). Antibiotics were then subjected to two-fold serial dilutions across the designated wells by transferring 50 µL between adjacent wells containing 50 µL Mueller–Hinton broth, with the final 50 µL discarded. Control antibiotic dilution series were prepared in parallel.

Following determination of the extract MIC, extracts were prepared at fixed sub-inhibitory concentrations corresponding to either 1/4 or 1/8 of their MIC. A volume of 50 µL of the appropriate extract concentration was added to each test well containing the serially diluted antibiotic, resulting in a constant extract concentration across the antibiotic dilution series. Thus, only the antibiotic concentration was serially diluted, whereas the extract concentration remained constant throughout the assay. For antibiotic control wells, 50 µL of sterile water was added in place of the extract. Extract controls containing the corresponding sub-MIC concentration (1/4 or 1/8 MIC) and 50 µL sterile water were also included.

Finally, 100 µL of an MRSA suspension (5 × 10^5^ CFU/mL) was added to each well, and plates were incubated at 37 °C for 18 h. Cell viability was determined using the INT colorimetric assay as described previously. Antibiotic modulation was classified as synergistic (FICI < 0.5) or additive (FICI = 0.5–1.0) according to established FICI criteria (Equation (1)) [91]. All experiments were performed using three independent biological replicates.

(1) $$\mathrm{FICI}=\frac{MIC\;Antibiotic\;combination}{MIC\;Antibiotic\;alone}+\frac{MIC\;extract\;combination}{MIC\;extract\;alone}$$

### 4.7. Time-Kill Kinetics

The time-kill kinetics of *P. canaliculata* (>30 kDa PEE) were evaluated both individually and in combination with penicillin against an MRSA isolate (HA 549). Experimental cultures were prepared by adding 8 mL of Mueller–Hinton broth containing an initial inoculum of 5 × 10^5^ CFU/mL. Four control conditions were included in the assay: (i) a negative control consisting of 1 mL sterile water and 1 mL culture; (ii) a penicillin control containing 1 mL sterile water and 1 mL penicillin at a subtherapeutic concentration; (iii) an extract control containing 1 mL sterile water and 1 mL of each extract at subtherapeutic concentration (1/4 MIC); and (iv) a penicillin MIC control containing 1 mL sterile water and 1 mL penicillin at its respective MIC. In addition, a modulation sample containing 1 mL penicillin and 1 mL extract (1/4 MIC), both at subtherapeutic concentrations, was also included. All cultures were agitated at 150 rpm and incubated in the dark at 37 °C for 16 h, in accordance with the European Committee on Antimicrobial Susceptibility (EUCAST) guidelines [92]. Samples were then collected at predetermined time points and analysed using the spread plate method (*n* = 3). Total viable counts were confirmed via serial dilution and plating on nutrient agar, followed by incubation for 24 h at 37 °C. The initial culture was Gram stained to ensure a pure culture.

### 4.8. Total Phenolics Assay

The total phenolic content of all extracts was evaluated using three technical and three biological replicates, as previously described by Kenny et al. [93]. Briefly, 100 μL of extract solubilised in methanol was added to a microcentrifuge tube, followed by 100 μL of methanol, 100 μL of Folin–Ciocalteu (2N) reagent, and 700 μL of sodium carbonate (20% *w*/*v*). For the blank control, 100 μL of methanol was used in replacement of extract. Each microcentrifuge tube was vortexed and incubated in the dark, at room temperature for 20 min. Following that, samples were centrifuged at 13,000 RPM for 3 min using a Sorvall ST4R Plus Centrifuge, and the absorbance measured at 735 nm using a spectrophotometer (Jenway 6300). A phloroglucinol calibration curve (10–200 μg/mL) was used to express the results as gallic acid equivalents (μg GAE/ mg DWE) following linear regression.

### 4.9. Total Fucoidan Assay

The total fucoidan content was evaluated as previously described by Ahmad et al. [94]. A standard curve was prepared from a fucose standard (10–300 μg/mL). Here, 154 μL of sample or standard was added to a test tube, to which 692 μL 85% sulfuric acid was added and homogenised. Contents were boiled at 100 °C in a water bath for 20 min and were subsequently cooled upon removal. After, 154 μL 3% (*w*/*v*) cysteine hydrochloride was added and homogenised (vortexed), followed by a 2 h incubation period at room temperature in the dark. Results were read on the spectrophotometer (Jenway 7415) at two wavelengths, which were subtracted from each other (λ 396 nm–λ 427 nm). The testing of two wavelengths excludes the presence of other interfering sugars to ensure a high level of methyl pentose detection [93]. The resulting value was multiplied by two to account for the total fucose content in the fucoidan sample, reflecting the presence of two fucose monomers per fucoidan unit. The final results were expressed as µg fucoidan/ mg dry weight extract (DWE) ± standard deviation following linear regression (*n* = 3).

### 4.10. Mechanism of Action for Antibiotic Modulation

Several assays and experiments were conducted to investigate the potential mechanisms of action for the observed antibiotic modulation activity for the extract–antibiotic combination yielding the lowest FIC value.

#### 4.10.1. Cell Viability Assay

The cell viability assay was used to determine cell envelope leakage, as previously described by Khamrai et al. [95] with minor modifications. *P. canaliculata* > 30 kDa PEE was solubilised in water, sonicated (Elma S180 sonicator (37 kHz), Elma Ultrasonic, Germany) for 10 min and centrifuged at 4000 rpm for 5 min using a Sorvall ST4R plus centrifuge. A single HA-549 MRSA colony was inoculated in Muller–Hinton broth and incubated at 37 °C until the mid-logarithmic stage was reached according to optical density (OD_600_) reading. Next, 800 μL of culture was added to five separate microcentrifuge tubes: a negative control, positive control, extract control (1/4 MIC), penicillin control and the modulation combination (*P. canaliculata* > 30 kDa PEE (1/4 MIC) and penicillin at the new MIC). The negative control contained the addition of 200 μL of sterile water, the positive control contained 100 μL of sterile water and 100 μL of daptomycin (MIC), the extract control contained 100 μL of *P. canaliculata* > 30 kDa PEE and 100 μL of sterile water, and the penicillin control contained 100 μL of penicillin (at the new MIC) in addition to 100 μL of sterile water, while the modulation combination contained 100 μL of *P. canaliculata* > 30 kDa PEE and 100 μL of penicillin (at the new MIC). Samples were incubated for 1 h at 37 °C under agitation at 150 rpm. Following incubation, samples were centrifuged at 10,000 rpm for 8 min, where the supernatant was discarded and the pellet resuspended in 800 μL of Ringers solution. After, 100 μL of 4′,6-diamidino-2-phenylindole (DAPI) solution (100 μg/mL) and 100 μL of propidium iodide (PI) (100 μg/mL) were added (final concentration 10 μg/mL) and incubated in the dark for 30 min at 37 °C under agitation (150 rpm). Following incubation, 10 μL of sample was analysed using a Canon EOS 1300D camera attached to Nikon Eclipse 80i for fluorescent microscopy. EOS utility software (version 3.7.0.0) was used for capturing images (×400 magnification) (ISO 100) with an ND4 filter applied to remove background florescence. All samples and controls were tested to yield 3 biological replicates.

#### 4.10.2. Biofilm Inhibition Assay

All MRSA isolates were initially evaluated for their ability to form strong biofilms (OD > 0.24), as previously defined by Pokhrel et al. [96]. Three HA-MRSAs and one CA-MRSA exhibited the strongest biofilm formation and were therefore selected to evaluate the extracts’ ability to inhibit biofilm development. A broth microdilution assay was performed using tryptic soy broth (TSB) supplemented with 1% (*w*/*v*) glucose, as previously described by Haney et al. [97]. Briefly, 200 µL of extract or fractions was added to the first lane of a sterile 96-well microtiter plate (*n* = 3 technical replicates). Two-fold serial dilutions of the extract or fractions were prepared across the plate using the TSB supplemented with 1% glucose (*w*/*v*) as the diluent. Following dilution, 100 µL of 5 x10^5^ CFU/mL bacterial culture in TSB supplemented with 1% (*w*/*v*) glucose was added to each well. Plates were incubated at 37 °C, and biofilm formation was assessed after 24 h of incubation, respectively (*n* = 3).

Following incubation, broth and planktonic cells were discarded by gently inverting the plates into a basin containing bleach, while all wells were washed thrice using Ringer’s solution (150 μL) to remove remaining broth. Ringer’s solution was gently discarded as mentioned above. Subsequently, 150 μL methanol was added to each well to fix the plates for 20 min. After removal, 150 μL of crystal violet solution (0.1% *v*/*v*) was added to each well and incubated for 30 min (37 °C). Following incubation, excess non-adherent crystal violet was removed using deionised water. After the residual water on the plates was dried (37 °C), 150 μL of acetic acid (33% *v*/*v*) was added to each well and incubated at 37 °C for 20 min to solubilise the crystal violet bound to the biofilm matrix. Plates were then analysed using FLUOstar OPTIMA (BMG LABTECH) at λ 640 nm. Acetic acid (33% *v*/*v*) control absorbance was deducted from each well. Results were calculated as % inhibition ± standard deviation, with reference to Equation (2).

(2) $$\%\;Biofilm\;inhibition=\frac{Negative\;control-Sample}{Negative\;control}\times 100$$

Equation (2)—Biofilm inhibition % formula.

#### 4.10.3. RNA Isolation

RNA-sequencing was carried out using the clinical MRSA isolate HA-549 against subtherapeutic concentrations of both penicillin (0.47 µg/mL) and *P. canaliculata* PEE > 30 kDa (1/12 MIC) to identify differentially expressed genes (DEGs). The *P. canaliculata* PEE > 30 kDa in combination with penicillin against the MRSA HA-549 isolate was selected due to the lowest observable FIC value (0.23), where 1/12 MIC of extract was selected due to its inhibitive effect on growth without compromising RNA extraction quality. Cultures were prepared as previously described by Wu et al. [98]. Briefly, overnight stationary-phase cultures were used to inoculate 8 mL of fresh Mueller–Hinton broth in 50 mL centrifuge tubes, in triplicate, to an initial OD_600_ of 0.01. Four conditions were evaluated: (i) a water control, (ii) a penicillin-only control, (iii) an extract control, and (iv) a modulation group containing both penicillin and extract. Cultures were incubated in the dark at 37 °C with agitation for approximately 3.5 h, until the mid-exponential phase (OD_600_ ≈ 0.45) was reached. At this point, treatments were applied as follows: the control received 2 mL sterile water; the penicillin control received 1 mL sterile water and 1 mL subtherapeutic penicillin; the extract control received 1 mL sterile water and 1 mL extract solubilised in sterile water; and the modulation group received 1 mL penicillin and 1 mL extract solubilised in sterile water, which were incubated for a further 30 min at 37 °C with agitation. Following incubation, 20 mL of RNAlater (Merck) was added to the culture and was further incubated at room temperature for 5 min, followed by centrifugation (8000× *g* for 5 min) and resuspension in 700 µL of RLT buffer (Qiagen RNeasy Mini Kit), which were subsequently transferred to lysis matrix B tubes (Fisher Scientific) for mechanical disruption using a FastPrep instrument (two cycles of 40 s at 6.5 m s^−1^). RNA was subsequently extracted following the manufacturer’s protocol for the Qiagen RNeasy Mini Kit. Extracted RNA was treated with DNase (Invitrogen Turbo DNase) to remove genomic DNA contamination.

#### 4.10.4. Transcriptomic Analysis

Samples were transported on dry ice to Novogene (UK) Company Limited, Cambridge, England. RNA quantity and quality metrics were assessed using a combination of agarose gel electrophoresis, Nanodrop, and Agilent 5400. Quality control (QC) analyses showed RNA samples had a median RNA Integrity Number (RIN) of 9.3, with a minimum RIN of 8.7. Subsequently, rRNA was depleted from the total RNA and then precipitated with ethanol. First-strand cDNA synthesis was performed using random hexamer primers. During second-strand cDNA synthesis, dUTPs were replaced by dTTPs. Strand-specific directional libraries were generated by end repair, A-tailing, and adaptor ligation. Libraries were sequenced on an Illumina NovaSeq X Plus, with paired-end 150 bp reads. Data quality post-sequencing was checked to ensure it met or exceeded industry standards, with ≥85% of bases with a Phred30 quality score. Differentially expressed genes (DEGs) were defined as log2 fold change ≥ ±1, *p* adj < 0.05 when compared to an additional sample or control. Kyoto Encyclopaedia of Genes and Genomes (KEGG) enrichment analysis indicated an enrichment of metabolic pathways. To improve dataset reliability and minimise the false positives commonly associated with transcriptomic analyses, adjusted *p*-values (*p* adj < 0.05) were used instead of raw p-values. A previously typed MRSA ST22 isolate was used for genomic reference (NCBI RefSeq assembly GCF_000695215.1) on the basis of being the most predominantly isolated MRSA isolate in Irish hospitals at the time of collection (2023) [99].

## 5. Conclusions

This study demonstrates the antimicrobial and β-lactam-modulating activity of extracts derived from *P. canaliculata*. Large-molecular-weight (>10 kDa) dialysed extracts, which were further fractionated into phlorotannin-enriched fractions, demonstrated moderate antimicrobial activity, and strong modulatory activity against a range of hospital-acquired and community-acquired MRSA isolates. Whole-transcriptome sequencing of a clinical MRSA isolate exposed to subtherapeutic concentrations of both penicillin and the *P. canaliculata* > 30 kDa phlorotannin-enriched extract has identified several differentially expressed genes, providing novel insight into the mechanisms underlying the observed modulatory effects, namely, accelerated autolysis, cell envelope disruption, inhibition of protein synthesis, reduced DNA replication, iron homeostasis disruption and decreased active transport. The use of antimicrobial combinations that act on multiple, mechanistically diverse targets may help reduce selective pressure and slow the development of antimicrobial resistance, while offering novel therapeutic options amid a limited antimicrobial pipeline. Further investigation using MRSA mutants corresponding to the identified differentially expressed genes is warranted to elucidate the underlying mechanisms of action, which may then be targeted for future antibiotic-modulating therapeutics. The modulation of first-line β-lactams against clinical infections could help preserve broad-spectrum, last-resort antibiotics for severe, multidrug-resistant cases.

Overall, this study highlights the capacity of aqueous extracts derived from *P. canaliculata* to enhance β-lactam efficacy against both hospital-acquired and community-acquired clinical MRSA isolates. In addition, these findings provide a valuable bioactivity framework for future investigations assessing molecular weight fractions derived from *P. canaliculata*. Collectively, the results support the continued exploration of marine natural products, namely macroalgae, as a promising source of antibiotic modulators against clinically relevant resistant pathogens.

## Figures and Tables

**Table 1:** The antimicrobial activity of *P. canaliculata* extracts, represented as MIC (*n* = 3). The initial ‘crude extract’ underwent dialysis, which resulted in a range of fractions expressed in kilodaltons (kDa). ‘-’, extracts were not active at the tested concentration of 2000 μg/mL; ‘CE’ = Crude Extract; ‘PEE’ = Phlorotannin Enriched Extract; ‘FEE’ = Fucoidan Enriched Extract.

Minimum Inhibitory Concentration (MIC) (μg/mL)														
MRSA Isolate	CE	<3 CE	3–10 CE	>10 CE	10–30 CE	>30 CE	>30 PEE	>30 FEE	30–100 CE	30–100 PEE	30–100 FEE	>100 CE	>100 PEE	>100 FEE
HA 1	1000	-	-	750	-	500	375	1000	187.5	375	-	750	750	-
HA 501	1500	-	-	750	-	375	250	1000	375	250	1000	750	500	-
HA 549	2000	-	-	750	-	500	375	-	375	250	-	750	500	-
HA 529	750	-	-	500	-	375	250	750	250	250	1000	750	500	-
CA 87	1500	-	-	500	-	500	375	1000	375	250	1000	750	500	1000
CA 254	750	-	-	500	-	500	375	1000	375	375	1000	750	750	-
CA 555	2000	-	-	750	-	500	375	-	375	375	1000	750	500	1000

**Table 2:** Condensed results displaying the antibiotic modulation of the crude extract and >30 kDa PEE tested at 1/4 MIC against HA-MRSA isolates, where the respective MIC under modulation is listed as ‘MICM’, in addition to the fold-reduction (x-FR) from old antibiotic MIC (*n* = 3). MIC and MICM concentrations expressed as µg/mL. A complete table of results can be seen in Table S5.

		HA 1			HA 501			HA 549			HA 529		
Antibiotic	Extract (kDa)	MIC	MICM	x-FR	MIC	MICM	x-FR	MIC	MICM	x-FR	MIC	MICM	x-FR
Amoxicillin	Crude	250	31.3	8.0	5	0.63	8.0	7.8	0.63	12.5	25	-	-
	>30 PEE		9.37	26.7		0.5	10.0		0.38	20.8		11.7	2.1
Ampicillin	Crude	250	31.3	8.0	5	1.56	3.2	3.9	0.31	12.5	25	6.25	4.0
	>30 PEE		9.37	26.7		0.5	10.0		0.38	10.4		7.81	3.2
Oxacillin	Crude	62.5	7.81	8.0	0.97	-	-	0.625	-	-	50	-	-
	>30 PEE		2.93	21.3		0.50	1.9		0.38	1.7		23.4	2.1
Penicillin	Crude	250	125	2.0	5	0.19	25.6	12.5	0.19	64.0	50	25.0	2.0
	>30 PEE		9.37	26.7		0.47	10.7		0.47	26.7		-	-

**Table 3:** Condensed results displaying the antibiotic modulation of the crude extract and >30 kDa PEE tested at 1/4 MIC against CA-MRSA isolates, where the respective MIC under modulation is listed as ‘MICM’, in addition to the fold-reduction (x-FR) from old antibiotic MIC (*n* = 3). MIC and MICM concentrations expressed as µg/mL. A complete table of results can be seen in Table S6.

		CA 87			CA 254			CA 555		
	Extract (kDa)	MIC	MICM	x-FR	MIC	MICM	x-FR	MIC	MICM	x-FR
Amoxicillin	Crude	25	3.13	8.0	25	3.13	8.0	3.13	-	-
	>30 PEE		0.75	33.3		0.38	66.7		0.38	8.3
Ampicillin	Crude	15.6	1.25	12.5	15.6	3.13	5.0	3.13	0.63	6.2
	>30 PEE		0.75	20.8		0.75	20.8		0.38	10.4
Oxacillin	Crude	12.5	-	-	15.6	5.0	3.1	0.97	-	-
	>30 PEE		1.88	6.7		1.25	12.5		0.38	2.6
Penicillin	Crude	15.6	0.78	20.0	25	3.13	8.0	12.5	0.39	32.0
	>30 PEE		3.13	5.0		9.37	2.7		0.63	20.0

**Table 4:** DEGs identified exclusively within the modulation complex (*P. canaliculata* > 30 kDa PEE and penicillin), which were not detected in any of the control samples, as they did not meet the inclusion criteria in those conditions (Log2FC +1/−1; *p* adj < 0.05). Some entries correspond to protein domain annotations (Pfam IDs) rather than individual genes, which are typically conserved. For these, the protein contains a domain similar to previously characterised proteins and likely has a related molecular function. Gene symbol/annotation represents the closest functional assignment available for each feature, including gene symbols for coding-DNA sequences (CDSs) and functional labels for non-coding RNAs.

Primary ID	ID Type	Gene/Annotation	Feature Type	Pfam Domain	Log2FC	*p* adj Value	Proposed Function
*cwrA*	Gene name	*cwrA*	CDS	-	+3.12	5.63 × 10^−15^	Cell envelope and antibiotic resistance
*yvcK*	Gene name	*yvcK*	CDS	-	+1.12	9.24 × 10^−4^	
*dltC*	Gene name	*dltC*	CDS	-	+1.01	2.89 × 10^−5^	
*dltD*	Gene name	*dltD*	CDS	-	+1.06	2.96 × 10^−7^	
*vraE*	Gene name	*vraE*	CDS	-	+1.31	1.49 × 10^−3^	
*uhpT*	Gene name	*uhpT*	CDS	-	+1.21	1.33 × 10^−6^	
*drp35*	Gene name	*drp35*	CDS	-	+1.60	8.32 × 10^−9^	
-	Pfam domain	PBP transpeptidase domain and PBP dimerisation domain	Protein domain	PF00905 PF03717	+2.39	1.41 × 10^−8^	
*lrgB*	Gene name	*lrgB*	CDS	-	+1.31	2.70 × 10^−5^	
*uspA*	Gene name	*uspA*	CDS	-	+1.21	5.91 × 10^−6^	
*rpsT*	Gene name	*rpsT*	CDS	-	−1.06	2.49 × 10^−5^	Protein synthesis disruption
*queH*	Gene name	*queH*	CDS	-	−1.00	2.13 × 10^−3^	
*catB*	Gene name	*catB*	CDS	-	+1.02	8.22 × 10^−3^	
*ssrA*	Gene name	*ssrA*	CDS	-	+1.03	1.04 × 10^−6^	
ER16_RS11455	Locus tag	tRNA-gln	tRNA	-	+1.17	2.84 × 10^−2^	
ER16_RS09700	Locus tag	tRNA-his	tRNA	-	+1.32	1.44 × 10^−2^	
*polC*	Gene name	*polC*	CDS	-	−1.09	4.14 × 10^−4^	DNA replication inhibition
ER16_RS12165	Pfam domain	HTH transcriptional regulator	Protein domain	PF12833	−1.36	4.32 × 10^−6^	
*ftnA*	Gene name	*ftnA*	CDS	-	−2.08	1.21 × 10^−22^	Iron homeostasis disruption
*fecCD*	Gene name	*fecCD*	CDS	-	+1.44	9.83 × 10^−18^	
*arcA*	Gene name	*arcA*	CDS	-	+2.40	2.55 × 10^−14^	
*yvcK*	Gene name	*yvcK*	CDS	-	+1.12	9.24 × 10^−4^	
*sdaAA*	Gene name	*sdaAA*	CDS	-	+1.23	7.43 × 10^−10^	
*ureC*	Gene name	*ureC*	CDS	-	+1.15	6.43 × 10^−4^	
*arcD*	Gene name	*arcD*	CDS	-	+1.33	1.31 × 10^−5^	
*hlgA*	Gene name	*hlgA*	CDS	-	+1.36	4.87 × 10^−9^	
*hlgB*	Gene name	*hlgB*	CDS	-	+1.53	4.51× 10^−5^	
*hlgC*	Gene name	*hlgC*	CDS	-	+1.73	2.78 × 10^−4^	
*sbnC*	Gene name	*sbnC*	CDS	-	+1.37	5.45 × 10^−3^	
ER16_RS03115	Pfam domain	ABC transporter	Protein domain	PF00950	−1.61	1.37 × 10^−11^	Active transport disruption
ER16_RS13360	Pfam domain	ABC transporter	Protein domain	PF12730	−1.32	1.61 × 10^−12^	
*yhaQ*	Gene name	*yhaQ*	CDS	-	−1.05	4.09 × 10^−4^	
*ydbJ*	Gene name	*ydbJ*	CDS	-	−1.57	8.13 × 10^−19^	
*sarA*	Gene name	*sarA*	CDS	-	−1.05	1.38 × 10^−11^	Autolytic activity
*arcD*	Gene name	*arcD*	CDS	-	+1.33	1.32 × 10^−5^	Metabolism shift
*ald*	Gene name	*ald*	CDS	-	−1.17	3.09 × 10^−6^	
*fadA*	Gene name	*fadA*	CDS	-	+1.03	6.45 × 10^−4^	
*carA*	Gene name	*carA*	CDS	-	+1.50	2.88 × 10^−2^	
*ureC*	Gene name	*ureC*	CDS	-	+1.15	6.43 × 10^−4^	
*hisA*	Gene name	*hisA*	CDS	-	+1.01	3.51 × 10^−2^	
*gntK*	Gene name	*gntK*	CDS	-	+1.23	1.89 × 10^−2^	
ER16_RS10730	Pfam domain	Nitroreductase	Protein domain	PF00881	−1.08	4.89 × 10^−7^	
ilvd	Gene name	ilvd	CDS	-	+1.32	1.74 × 10^−2^	
mntC	Gene name	mntC	CDS	-	−1.40	3.31 × 10^−15^	
mntB	Gene name	mntB	CDS	-	−1.84	1.95 × 10^−8^	
-	Pfam domain	Zinc uptake protein	Protein domain	PF01297	−1.44	3.26 × 10^−5^	
-	Pfam domain	SMP-30/Gluconolactonase	Protein domain	PF08450	+2.44	4.93 × 10^−7^	
-	Pfam domain	GAF domain and Nitrate reductase gamma subunit	Protein domain	PF13185 PF02665	+1.66	2.64 × 10^−5^	
ER16_RS00720	Pfam domain	Nickel responsive protein	Protein domain	PF14026	+1.32	1.67 × 10^−4^	
ER16_RS03130	Pfam domain	M50 metallopeptidase	Protein domain	PF13398	+1.10	1.54 × 10^−2^	
ER16_RS01015	Pfam domain	Acyl-coA dehydrogenase family domains	Protein domain	PF00441 PF02770	+1.35	1.94 × 10^−2^	
-	Pfam domain	Isocitrate dehydrogenase Citrate synthase	Protein domain	PF00180 PF00285	+1.03	4.01 × 10^−3^	
ER16_RS08545	Pfam domain	Unknown function	Protein domain	PF16284	+1.98	3.34 × 10^−16^	Unknown
-	Pfam domain	Unknown function	Protein domain	PF17412	+3.44	5.05 × 10^−21^	
-	Pfam domain	Unknown function	Protein domain	PF07274	+1.17	6.85 × 10^−3^	
ER16_RS01045	Pfam domain	Unknown function	Protein domain	P76243	−1.38	3.71 × 10^−4^	

## Data Availability

All data are included in the present work and are available from the corresponding author upon reasonable request.

## Supplementary Materials

The following supporting information can be downloaded at https://www.mdpi.com/article/10.3390/md24080280/s1, Table S1: Total fucoidan and total phloroglucinol content (present in tested dry weight extracts (DWE) of *P. canaliculata*; Tables S2–S6: Antibiotic modulating activity of *P. canaliculata* extracts against MRSA isolates; Table S7: Additional genes with altered expression (*p* adj < 0.05) in the treatment complex not identified in the control parameters.

## Abbreviations

Adenosine triphosphate (ATP), American Type Culture Collection (ATCC), antimicrobial resistance (AMR), ATP-binding cassette (ABC), colony forming unit (CFU), community-acquired (CA), complementary deoxyribonucleic acid (cDNA), degree of polymerisation (DP), deoxyribose nucleic acid (DNA), deoxythymidine triphosphate (dTTP), deoxyuridine triphosphate (dUTP), differentially expressed genes (DEGs), dry-weight extract (DWE), electron ionisation (EI), European Union (EU), gene oncology (GO), hospital-acquired (HA), interquartile range (IQR), iodonitrotetrazolium chloride (INT), Kyoto Encyclopedia of Genes and Genomes (KEGG), methicillin-resistant *Staphylococcus aureus* (MRSA), methicillin-sensitive *Staphylococcus aureus* (MSSA), milligram/millilitre (mg/mL), minimum inhibitory concentration (MIC), molecular weight cut-off (MWCO), penicillin-binding proteins (PBPs), phlorotannin-enriched extract(s) (PEE), phloroglucinol equivalent (PGE), quality control (QC), revolutions per minute (RPM), ribonucleic acid (RNA), RNA integrity number (RIN), tricarboxylic acid (TCA), triple axis detector (TAD), volume/volume (*v*/*v*), weight/volume (*w*/*v*), and zonal inhibition (ZI).

## References

[1] Murray C.J.L., Ikuta K.S., Sharara F., Swetschinski L., Aguilar G.R., Gray A., Han C., Bisignano C., Rao P., Wool E. (2022). Global burden of bacterial antimicrobial resistance in 2019: A systematic analysis. Lancet.

[2] Mestrovic T., Aguilar G.R., Swetschinski L.R., Ikuta K.S., Gray A.P., Weaver N.D., Han C., Wool E.E., Hayoon A.G., Hay S.I. (2022). The burden of bacterial antimicrobial resistance in the WHO European region in 2019: A cross-country systematic analysis. Lancet Public Health.

[3] ECDC (2023). Antimicrobial Consumption in the EU/EEA (ESAC-Net)—Annual Epidemiological Report for 2023.

[4] Poudel A.N., Zhu S., Cooper N., Little P., Tarrant C., Hickman M., Yao G. (2023). The economic burden of antibiotic resistance: A systematic review and meta-analysis. PLoS ONE.

[5] Cassini A., Högberg L.D., Plachouras D., Quattrocchi A., Hoxha A., Simonsen G.S., Colomb-Cotinat M., Kretzschmar M.E., Devleesschauwer B., Cecchini M. (2019). Attributable deaths and disability-adjusted life-years caused by infections with antibiotic-resistant bacteria in the EU and the European Economic Area in 2015: A population-level modelling analysis. Lancet Infect. Dis..

[6] O’Neill J. (2016). Tackling Drug-Resistant Infections Globally: Final Report and Recommendations.

[7] Lim D., Strynadka N.C.J. (2002). Structural basis for the β lactam resistance of PBP2a from methicillin-resistant Staphylococcus aureus. Nat. Struct. Biol..

[8] Kluytmans-VandenBergh M.F.Q., Kluytmans J. (2006). Community-acquired methicillin-resistant Staphylococcus aureus: Current perspectives. Clin. Microbiol. Infect..

[9] Aetrugh S., Aboshkiwa M., Husien W., Erhuma M., Corrente M., Grandolfo E., Ellabib M., Emahbes T., Mustafa M. (2017). Antimicrobial resistance profile and molecular characterization of methicillin-resistant staphylococcus isolates in Tripoli Central Hospital, Libya. Libyan Int. Med. Univ. J..

[10] Sauer K., Stoodley P., Goeres D.M., Hall-Stoodley L., Burmølle M., Stewart P.S., Bjarnsholt T. (2022). The biofilm life cycle: Expanding the conceptual model of biofilm formation. Nat. Rev. Microbiol..

[11] Assefa M., Amare A. (2022). Biofilm-associated multi-drug resistance in hospital-acquired infections: A review. Infect. Drug Resist..

[12] Lewis K. (2008). Multidrug tolerance of biofilms and persister cells. Bact. Biofilms.

[13] Shin H.-J., Yang S., Lim Y. (2021). Antibiotic susceptibility of Staphylococcus aureus with different degrees of biofilm formation. J. Anal. Sci. Technol..

[14] Parastan R., Kargar M., Solhjoo K., Kafilzadeh F. (2020). Staphylococcus aureus biofilms: Structures, antibiotic resistance, inhibition, and vaccines. Gene Rep..

[15] Wu H.R., Jia C., Wang X.H., Shen J., Tan J.L., Wei Z.Y., Wang S.L., Sun D., Xie Z., Luo F. (2023). The impact of methicillin resistance on clinical outcome among patients with Staphylococcus aureus osteomyelitis: A retrospective cohort study of 482 cases. Sci. Rep..

[16] Hirabayashi A., Yahara K., Oka K., Kajihara T., Ohkura T., Hosaka Y., Shibayama K., Sugai M., Yagi T. (2024). Comparison of disease and economic burden between MRSA infection and MRSA colonization in a university hospital: A retrospective data integration study. Antimicrob. Resist. Infect. Control.

[17] Adeiza S.S., Aminul I. (2024). Meta-meta-analysis of the mortality risk associated with MRSA compared to MSSA bacteraemia. Le. Infez. Med..

[18] WHO (2023). 2023 Antibacterial Agents in Clinical and Preclinical Development: An Overview and Analysis.

[19] Butler M.S., Gigante V., Sati H., Paulin S., Al-Sulaiman L., Rex J.H., Fernandes P., Arias C.A., Paul M., Thwaites G.E. (2022). Analysis of the clinical pipeline of treatments for drug-resistant bacterial infections: Despite progress, more action is needed. Antimicrob. Agents Chemother..

[20] Catarino M.D., Pires S.M.G., Silva S., Costa F., Braga S.S., Pinto D.C.G.A., Silva A.M.S., Cardoso S.M. (2022). Overview of phlorotannins’ constituents in Fucales. Mar. Drugs.

[21] Lee D.-S., Kang M.-S., Hwang H.-J., Eom S.-H., Yang J.-Y., Lee M.-S., Lee W.-J., Jeon Y.-J., Choi J.-S., Kim Y.-M. (2008). Synergistic effect between dieckol from Ecklonia stolonifera and β-lactams against methicillin-resistant Staphylococcus aureus. Biotechnol. Bioprocess Eng..

[22] Meshalkina D., Tsvetkova E., Orlova A., Islamova R., Grashina M., Gorbach D., Babakov V., Francioso A., Birkemeyer C., Mosca L. (2023). First insight into the neuroprotective and antibacterial effects of phlorotannins isolated from the cell walls of brown algae Fucus vesiculosus and *Pelvetia canaliculata*. Antioxidants.

[23] Kopf A.H., Hammer A., Dirks R.A.M., Araya-Cloutier C. (2025). Antimicrobial activity of North Atlantic Ocean macroalgae and the size-activity relationships of phlorotannins in Fucus vesiculosus. Algal Res..

[24] Birkemeyer C., Lemesheva V., Billig S., Tarakhovskaya E. (2020). Composition of intracellular and cell wall-bound phlorotannin fractions in fucoid algae indicates specific functions of these metabolites dependent on the chemical structure. Metabolites.

[25] Hosseini S.V., Dastgerdi H.E., Tahergorabi R. (2024). Marine mannitol: Extraction, structures, properties, and applications. Processes.

[26] Jayaratne P., Rutherford C. (1999). Detection of methicillin-resistant Staphylococcus aureus (MRSA) from growth on mannitol salt oxacillin agar using PCR for nosocomial surveillance. Diagn. Microbiol. Infect. Dis..

[27] Lee J.-H., Eom S.-H., Lee E.-H., Jung Y.-J., Kim H.-J., Jo M.-R., Son K.-T., Lee H.-J., Kim J.H., Lee M.-S. (2014). In vitro antibacterial and synergistic effect of phlorotannins isolated from edible brown seaweed Eisenia bicyclis against acne-related bacteria. Algae.

[28] Eom S.-H., Lee D.-S., Jung Y.-J., Park J.-H., Choi J.-I., Yim M.-J., Jeon J.-M., Kim H.-W., Son K.-T., Je J.-Y. (2014). The mechanism of antibacterial activity of phlorofucofuroeckol-A against methicillin-resistant Staphylococcus aureus. Appl. Microbiol. Biotechnol..

[29] Lopes G., Sousa C., Silva L.R., Pinto E., Andrade P.B., Bernardo J., Mouga T., Valentão P. (2012). Can phlorotannins purified extracts constitute a novel pharmacological alternative for microbial infections with associated inflammatory conditions?. PLoS ONE.

[30] Tang J., Wang W., Chu W. (2020). Antimicrobial and anti-quorum sensing activities of phlorotannins from seaweed (*Hizikia fusiforme*). Front. Cell. Infect. Microbiol..

[31] Saxena D., Maitra R., Bormon R., Czekanska M., Meiers J., Titz A., Verma S., Chopra S. (2023). Tackling the outer membrane: Facilitating compound entry into Gram-negative bacterial pathogens. npj Antimicrob. Resist..

[32] Liu J., Huang T., Xu Z., Mao Y., Soteyome T., Liu G., Qu C., Yuan L., Ma Q., Zhou F. (2023). Sub-MIC streptomycin and tetracycline enhanced Staphylococcus aureus Guangzhou-SAU749 biofilm formation, an in-depth study on transcriptomics. Biofilm.

[33] Ranieri M.R.M., Whitchurch C.B., Burrows L.L. (2018). Mechanisms of biofilm stimulation by subinhibitory concentrations of antimicrobials. Curr. Opin. Microbiol..

[34] Besednova N.N., Andryukov B.G., Zaporozhets T.S., Kryzhanovsky S.P., Kuznetsova T.A., Fedyanina L.N., Makarenkova I.D., Zvyagintseva T.N. (2020). Algae polyphenolic compounds and modern antibacterial strategies: Current achievements and immediate prospects. Biomedicines.

[35] Bronner S., Monteil H., Prévost G. (2004). Regulation of virulence determinants in Staphylococcus aureus: Complexity and applications. FEMS Microbiol. Rev..

[36] Dunman P.Á., Murphy E., Haney S., Palacios D., Tucker-Kellogg G., Wu S., Brown E.L., Zagursky R.J., Shlaes D., Projan S.J. (2001). Transcription profiling-based identification of Staphylococcus aureus genes regulated by the agr and/or sarA loci. J. Bacteriol..

[37] Beenken K.E., Blevins J.S., Smeltzer M.S. (2003). Mutation of sarA in Staphylococcus aureus limits biofilm formation. Infect. Immun..

[38] Tsang L.H., Cassat J.E., Shaw L.N., Beenken K.E., Smeltzer M.S. (2008). Factors contributing to the biofilm-deficient phenotype of Staphylococcus aureus sarA mutants. PLoS ONE.

[39] Beenken K.E., Mrak L.N., Griffin L.M., Zielinska A.K., Shaw L.N., Rice K.C., Horswill A.R., Bayles K.W., Smeltzer M.S. (2010). Epistatic relationships between sarA and agr in Staphylococcus aureus biofilm formation. PLoS ONE.

[40] Fujimoto D.F., Bayles K.W. (1998). Opposing roles of the Staphylococcus aureus virulence regulators, Agr and Sar, in Triton X-100-and penicillin-induced autolysis. J. Bacteriol..

[41] Li Y., Yuan S., Yan P., Zhai S., He Z., Su H., Zhu Z., He Q., Xu W., Sun B. (2026). Staphylococcal accessory regulator SarA-mediated modulation of autolysis and surface charge enables Staphylococcus aureus to evade vancomycin killing. Msystems.

[42] Li J., Lu T., Chu Y., Zhang Y., Zhang J., Fu W., Sun J., Liu Y., Liao X.P., Zhou Y.F. (2024). Cinnamaldehyde targets SarA to enhance β-lactam antibiotic activity against methicillin-resistant Staphylococcus aureus. Mlife.

[43] Wang G., Li L., Wang X., Li X., Zhang Y., Yu J., Jiang J., You X., Xiong Y.Q. (2019). Hypericin enhances β-lactam antibiotics activity by inhibiting sarA expression in methicillin-resistant Staphylococcus aureus. Acta Pharm. Sin. B.

[44] Balibar C.J., Shen X., McGuire D., Yu D., McKenney D., Tao J. (2010). CwrA, a gene that specifically responds to cell wall damage in Staphylococcus aureus. Microbiology.

[45] Gorke B., Foulquier E., Galinier A. (2005). YvcK of Bacillus subtilis is required for a normal cell shape and for growth on Krebs cycle intermediates and substrates of the pentose phosphate pathway. Microbiology.

[46] Foulquier E., Galinier A. (2017). YvcK, a protein required for cell wall integrity and optimal carbon source utilization, binds uridine diphosphate-sugars. Sci. Rep..

[47] Olchowik-Grabarek E., Sekowski S., Bitiucki M., Dobrzynska I., Shlyonsky V., Ionov M., Burzynski P., Roszkowska A., Swiecicka I., Abdulladjanova N. (2020). Inhibition of interaction between Staphylococcus aureus α-hemolysin and erythrocytes membrane by hydrolysable tannins: Structure-related activity study. Sci. Rep..

[48] Morikawa K., Hidaka T., Murakami H., Hayashi H., Ohta T. (2005). Staphylococcal Drp35 is the functional counterpart of the eukaryotic PONs. FEMS Microbiol. Lett..

[49] Groicher Kajetan H., Firek Brian A., Fujimoto David F., Bayles Kenneth W. (2000). The Staphylococcus aureus lrgAB Operon Modulates Murein Hydrolase Activity and Penicillin Tolerance. J. Bacteriol..

[50] Beltrame C.O., Cortes M.F., Bonelli R.R., Côrrea A.B.D.A., Botelho A.M.N., Americo M.A., Fracalanzza S.E.L., Figueiredo A.M.S. (2015). Inactivation of the autolysis-related genes lrgB and yycI in Staphylococcus aureus increases cell lysis-dependent eDNA release and enhances biofilm development in vitro and in vivo. PLoS ONE.

[51] Song Y., Lunde C.S., Benton B.M., Wilkinson B.J. (2012). Further insights into the mode of action of the lipoglycopeptide telavancin through global gene expression studies. Antimicrob. Agents Chemother..

[52] Hiron A., Falord M., Valle J., Débarbouillé M., Msadek T. (2011). Bacitracin and nisin resistance in Staphylococcus aureus: A novel pathway involving the BraS/BraR two-component system (SA2417/SA2418) and both the BraD/BraE and VraD/VraE ABC transporters. Mol. Microbiol..

[53] Xu S., Fu Z., Zhou Y., Liu Y., Xu X., Wang M. (2017). Mutations of the Transporter Proteins GlpT and UhpT Confer Fosfomycin Resistance in Staphylococcus aureus. Front. Microbiol..

[54] Wei Y., Liu Q., Xu C., Yu J., Zhao L., Guo Q. (2016). Damage to the membrane permeability and cell death of Vibrio parahaemolyticus caused by phlorotannins with low molecular weight from Sargassum thunbergii. J. Aquat. Food Product. Technol..

[55] Guo C., Wang C., Chen Q., Zheng S.H., Zhang F., Yan J., Long H.B., Luo J., Xuan X., Wang P. (2025). The dltC gene contributes to polyhexamethylene biguanide resistance in Staphylococcus aureus. Front. Microbiol..

[56] Clemans D.L., Kolenbrander P.E., Debabov D.V., Zhang Q., Lunsford R.D., Sakone H., Whittaker C.J., Heaton M.P., Neuhaus F.C. (1999). Insertional inactivation of genes responsible for thed-alanylation of lipoteichoic acid in Streptococcus gordonii DL1 (Challis) affects intrageneric coaggregations. Infect. Immun..

[57] Lin J., Zhou D., Steitz T.A., Polikanov Y.S., Gagnon M.G. (2018). Ribosome-targeting antibiotics: Modes of action, mechanisms of resistance, and implications for drug design. Annu. Rev. Biochem..

[58] Melnikov S., Ben-Shem A., Garreau de Loubresse N., Jenner L., Yusupova G., Yusupov M. (2012). One core, two shells: Bacterial and eukaryotic ribosomes. Nat. Struct. Mol. Biol..

[59] Knöppel A., Näsvall J., Andersson D.I. (2016). Compensating the fitness costs of synonymous mutations. Mol. Biol. Evol..

[60] Rydén-Aulin M., Shaoping Z., Kylsten P., Isaksson L.A. (1993). Ribosome activity and modification of 16S RNA are influenced by deletion of ribosomal protein S20. Mol. Microbiol..

[61] Natori Y., Nanamiya H., Akanuma G., Kosono S., Kudo T., Ochi K., Kawamura F. (2007). A fail-safe system for the ribosome under zinc-limiting conditions in Bacillus subtilis. Mol. Microbiol..

[62] Karzai A.W., Roche E.D., Sauer R.T. (2000). The SsrA–SmpB system for protein tagging, directed degradation and ribosome rescue. Nat. Struct. Biol..

[63] Frees D., Qazi S.N.A., Hill P.J., Ingmer H. (2003). Alternative roles of ClpX and ClpP in Staphylococcus aureus stress tolerance and virulence. Mol. Microbiol..

[64] Li Q., Zallot R., MacTavish B.S., Montoya A., Payan D.J., Hu Y., Gerlt J.A., Angerhofer A., de Crécy-Lagard V., Bruner S.D. (2021). Epoxyqueuosine reductase QueH in the biosynthetic pathway to tRNA queuosine is a unique metalloenzyme. Biochemistry.

[65] Ibrahim N., Yaacob W.A. (2017). Disruption of methicillin-resistant Staphylococcus aureus protein synthesis by tannins. Germs.

[66] Wang K., Chen J., Li W., Wang M., Fan T., Bu D., Ye S. (2025). Structural basis for antibiotic resistance by chloramphenicol acetyltransferase type A in Staphylococcus aureus. Sci. Rep..

[67] McHenry C.S. (2011). Breaking the rules: Bacteria that use several DNA polymerase IIIs. EMBO Rep..

[68] Lahiri I., Mukherjee P., Pata J.D. (2013). Kinetic characterization of exonuclease-deficient Staphylococcus aureus PolC, a C-family replicative DNA polymerase. PLoS ONE.

[69] Hamady A.B., Abd El-Fadeal N.M., Imbaby S., Nassar H.M.A., Sakr M.G., Marei Y.E. (2024). Expression of norA, norB and norC efflux pump genes mediating fluoroquinolones resistance in MRSA isolates. J. Infect. Dev. Ctries..

[70] Bolt E.L., Jenkins T., Russo V.M., Ahmed S., Cavey J., Cass S.D. (2016). Identification of Escherichia coli ygaQ and rpmG as novel mitomycin C resistance factors implicated in DNA repair. Biosci. Rep..

[71] Evans R.J., Davies D.R., Bullard J.M., Christensen J., Green L.S., Guiles J.W., Pata J.D., Ribble W.K., Janjic N., Jarvis T.C. (2008). Structure of PolC reveals unique DNA binding and fidelity determinants. Proc. Natl. Acad. Sci. USA.

[72] Chung K.T., Lu Z., Chou M.W. (1998). Mechanism of inhibition of tannic acid and related compounds on the growth of intestinal bacteria. Food Chem. Toxicol..

[73] Gal Y., Marcus H., Mamroud E., Aloni-Grinstein R. (2023). Mind the gap—A perspective on strategies for protecting against bacterial infections during the period from infection to eradication. Microorganisms.

[74] Stefanova D., Raychev A., Arezes J., Ruchala P., Gabayan V., Skurnik M., Dillon B.J., Horwitz M.A., Ganz T., Bulut Y. (2017). Endogenous hepcidin and its agonist mediate resistance to selected infections by clearing non–transferrin-bound iron. Blood J. Am. Soc. Hematol..

[75] Wang T., Jónsdóttir R.S., Liu H., Gu L., Kristinsson H.G., Raghavan S., Ólafsdóttir G.N. (2012). Antioxidant capacities of phlorotannins extracted from the brown algae Fucus vesiculosus. J. Agric. Food Chem..

[76] Frešer F., Bren U., Hostnik G. (2024). Chelation of iron (II) ions by ellagitannins—Effects of hexahydroxydiphenoyl and nonahydroxytriphenoyl groups. Spectrochim. Acta Part A Mol. Biomol. Spectrosc..

[77] Sheldon J.R., Marolda C.L., Heinrichs D.E. (2014). TCA cycle activity in S taphylococcus aureus is essential for iron-regulated synthesis of staphyloferrin A, but not staphyloferrin B: The benefit of a second citrate synthase. Mol. Microbiol..

[78] Nizam S.A., Zhu J., Ho P.Y., Shimizu K. (2009). Effects of arcA and arcB genes knockout on the metabolism in Escherichia coli under aerobic condition. Biochem. Eng. J..

[79] Friedman D.B., Stauff D.L., Pishchany G., Whitwell C.W., Torres V.J., Skaar E.P. (2006). Staphylococcus aureus redirects central metabolism to increase iron availability. PLoS Pathog..

[80] Huang S.-C., Burne R.A., Chen Y.-Y.M. (2014). The pH-dependent expression of the urease operon in Streptococcus salivarius is mediated by CodY. Appl. Environ. Microbiol..

[81] Sakanaka A., Kuboniwa M., Takeuchi H., Hashino E., Amano A. (2015). Arginine-Ornithine Antiporter ArcD Controls Arginine Metabolism and Interspecies Biofilm Development of Streptococcus gordonii. J. Biol. Chem..

[82] Beetham C.M., Schuster C.F., Kviatkovski I., Santiago M., Walker S., Gründling A. (2024). Histidine transport is essential for the growth of Staphylococcus aureus at low pH. PLoS Pathog..

[83] Wu H., Zhang Y., Li L., Li Y., Yuan L., Qiao J. (2022). Positive regulation of the DLT operon by TCSR7 enhances acid tolerance of Lactococcus lactis F44. J. Dairy Sci..

[84] Horsburgh M.J., Ingham E., Foster S.J. (2001). In Staphylococcus aureus, Fur is an interactive regulator with PerR, contributes to virulence, and is necessary for oxidative stress resistance through positive regulation of catalase and iron homeostasis. J. Bacteriol..

[85] Haley K.P., Skaar E.P. (2012). A battle for iron: Host sequestration and Staphylococcus aureus acquisition. Microbes Infect..

[86] Tanaka K.J., Song S., Mason K., Pinkett H.W. (2018). Selective substrate uptake: The role of ATP-binding cassette (ABC) importers in pathogenesis. Biochim. Et Biophys. Acta (BBA)-Biomembr..

[87] Otarigho B., Duffin P.M., Falade M.O. (2025). Natural Compounds Targeting ABC Transporters and MecA to Combat MRSA Antibiotic Resistance. Preprints.

[88] Zayed A., Muffler K., Hahn T., Rupp S., Finkelmeier D., Burger-Kentischer A., Ulber R. (2016). Physicochemical and biological characterization of fucoidan from Fucus vesiculosus purified by dye affinity chromatography. Mar. Drugs.

[89] Smyth T., Ramachandran V.N., Smyth W.F. (2009). A study of the antimicrobial activity of selected naturally occurring and synthetic coumarins. Int. J. Antimicrob. Agents.

[90] Embaby M.A., El-Raey M.A., Zaineldain M., Almaghrabi O., Marrez D.A. (2021). Synergistic effect and efflux pump inhibitory activity of Ficus nitida phenolic extract with tetracycline against some pathogenic bacteria. Toxin Rev..

[91] Odds F.C. (2003). Synergy, antagonism, and what the chequerboard puts between them. J. Antimicrob. Chemother..

[92] The European Committee on Antimicrobial Susceptibility Testing (2026). Breakpoint Tables for Interpretation of MICs and Zone Diameters.

[93] Kenny O., Smyth T.J., Hewage C.M., Brunton N.P. (2013). Antioxidant properties and quantitative UPLC-MS analysis of phenolic compounds from extracts of fenugreek (*Trigonella foenum-graecum*) seeds and bitter melon (*Momordica charantia*) fruit. Food Chem..

[94] Ahmad T.B.S. (2015). Methods for quantification and extraction of fucoidan, and quantification of the release of total carbohydrate and fucoidan from the brown algae Laminaria hyperborea. Norwegian Research Information Repository.

[95] Khamrai M., Banerjee S.L., Paul S., Samanta S., Kundu P.P. (2019). Curcumin entrapped gelatin/ionically modified bacterial cellulose based self-healable hydrogel film: An eco-friendly sustainable synthesis method of wound healing patch. Int. J. Biol. Macromol..

[96] Pokhrel S., Sharma N., Aryal S., Khadka R., Thapa T.B., Pandey P., Joshi G. (2024). Detection of Biofilm Production and Antibiotic Susceptibility Pattern among Clinically Isolated Staphylococcus aureus. J. Pathog..

[97] Haney E.F., Trimble M.J., Hancock R.E.W. (2021). Microtiter plate assays to assess antibiofilm activity against bacteria. Nat. Protoc..

[98] Wu J., NicAogáin K., McAuliffe O., Jordan K., O’Byrne C. (2022). Phylogenetic and phenotypic analyses of a collection of food and clinical Listeria monocytogenes isolates reveal loss of function of sigma B from several clonal complexes. Appl. Environ. Microbiol..

[99] Brennan G., Fleming T., Grier P., Fadejeva L., Eakins J., O’Connell B. (2023). National Meticillin-Resistant Staphylococcus aureus Reference Laboratory: Annual Report.

