# Supplementary material for: Transcriptomic Responses of MRSA to Penicillin-Modulating Phlorotannins Derived from Pelvetia canaliculata
Source: Mar Drugs. 2026 Aug 12;24(8):280. doi: 10.3390/md24080280 (PMC13514743; doi:10.3390/md24080280)
Supplement: Supplementary file 1 [file marinedrugs-24-00280-s001.zip › marinedrugs-4437185-supplementary.pdf]

Table S1 - Total fucoidan content (µg of fucose (x2)/mg DWE) and total phloroglucinol equivalents (µg PGE/mg DWE) (± standard deviation) present in tested dry weight extracts (DWE) of *P. canaliculata* (n = 3). Evaluated extracts of varying molecular weight ranges included crude extracts (CE), phlorotannin enriched extracts (PEE) and fucoidan enriched extracts (FEE). A notable increase in total phloroglucinol was evident in the majority of PEE fractions when compared to both the crude counterpart and the FEE. Similarly, total fucoidan content increased in FEE extracts, indicating optimal enrichment of these analytes from the crude extract.

Extract (kDa)	Total Fucoidan (µg/mg DWE)	Total Phloroglucinol (µg PGE/mg DWE)
**Crude**	60.95 ± 7.54	32.21 ± 2.34
**< 3 CE**	0.49 ± 1.32	14.29 ± 2.19
**3 – 10 CE**	4.63 ± 2.14	12.21 ± 1.42
**>10 CE**	78.4 ± 14.96	51.96 ± 2.21
**10 – 30 CE**	5.92 ± 3.10	16.8 ± 1.86
**>30 CE**	67.61 ± 19.94	46.74 ± 1.58
**>30 PEE**	25.25 ± 15.04	160.89 ± 3.67
**> 30 FEE**	169.87 ± 23.74	13.10 ± 2.12
**30 – 100**	3.59 ± 0.52	92.87 ± 5.48
**30 – 100 PEE**	9.75 ± 3.05	90.76 ± 2.0
**30 – 100 FEE**	27.99 ± 7.88	27.06 ± 1.78
**> 100 CE**	80.24 ± 17.24	27.92 ± 1.64
**>100 PEE**	20.78 ± 3.46	191.42 ± 1.06
**>100 FEE**	264.03 ± 17.73	14.44 ± 1.96

Table S2 - β-lactam-extract modulation, where A = initial antibiotic MIC; B = antibiotic MIC under modulation using 1/4 extract MIC; C = antibiotic MIC under modulation using 1/8 extract MIC, expressed as μg/mL. Both 'b' and 'c' FIC values correspond to the respective values seen in B and C, respectively (n=3).

Antibiotic	Isolate	>10 kDa					> 30 kDa				30 – 100 kDa				>100 kDa			
		MIC			FIC		MIC		FIC		MIC		FIC		MIC		FIC	
		A	B	C	b	c	B	C	b	c	B	C	b	c	B	C	b	c
Amoxicillin	HA 1	250	62.5	37.5	0.50	0.28	4.68	37.5	0.27	0.28	3.90	-	0.27	-	3.90	50	0.26	0.33
	HA 501	5	0.31	1.87	0.31	0.50	0.37	1.87	0.32	0.50	0.75	2.5	0.40	0.63	0.37	1.87	0.32	0.50
	HA 549	7.8	0.72	1.25	0.34	0.29	0.37	1.25	0.30	0.29	0.37	1.87	0.30	0.36	0.37	1.25	0.29	0.29
	HA 529	25	9.37	-	0.62	-	5.86	-	0.48	-	7.81	-	0.56	-	5.86	-	0.48	-
	CA 87	25	1.56	9.37	0.31	0.50	0.75	9.37	0.28	0.50	0.75	12.5	0.28	0.63	1.17	12.5	0.29	0.63
	CA 254	25	1.56	9.37	0.31	0.50	0.75	12.5	0.28	0.63	0.75	1205	0.28	48.33	0.5	12.5	0.27	0.63
	CA 555	3.13	0.39	-	0.37	-	0.37	-	0.37	-	0.37	-	0.37	-	0.37	-	0.36	-
Ampicillin	HA 1	250	62.5	50	0.50	0.33	4.68	37.5	0.27	0.28	5.84	-	0.27	-	4.68	37.5	0.27	0.28
	HA 501	5	0.31	1.25	0.31	0.38	0.37	1.25	0.32	0.38	0.5	2.5	0.35	0.63	0.37	1.25	0.32	0.38
	HA 549	3.9	0.31	1.25	0.33	0.45	0.31	0.93	0.33	0.36	0.37	1.87	0.34	0.60	0.37	0.93	0.34	0.36
	HA 529	25	6.25	-	0.50	-	9.37	-	0.62	-	-	-	-	-	-	-	-	-
	CA 87	15.6	1.56	9.37	0.35	0.73	0.37	9.37	0.27	0.73	0.75	9.37	0.30	0.73	1.17	12.5	0.33	0.93
	CA 254	15.6	1.56	9.37	0.35	0.73	0.37	9.37	0.27	0.73	0.75	12.5	0.30	0.93	-	12.5	-	0.93
	CA 555	3.9	0.39	0.5	0.35	0.25	0.25	0.75	0.31	0.32	0.37	-	0.34	-	0.37	0.75	0.34	0.32
Oxacillin	HA 1	62.5	1.95	31.2	0.28	0.62	2.93	31.2	0.30	0.62	1.95	31.25	0.28	0.63	1.17	23.4	0.25	0.50
	HA 501	0.97	0.31	-	0.57	-	0.5	-	0.77	-	0.12	-	0.37	-	0.5	-	0.77	-
	HA 549	0.63	0.15	-	0.49	-	-	-	-	-	-	-	-	-	-	-	-	-
	HA 529	50	15.6	-	0.56	-	11.7	-	0.48	-	23.4	-	0.72	-	11.7	-	0.48	-
	CA 87	12.5	-	-	-	-	1.25	-	0.35	-	1.87	-	0.40	-	2.5	-	0.45	-
	CA 254	15.6	1.25	-	0.33	-	1.25	-	0.33	-	1.87	-	0.37	-	1.87	-	0.37	-
	CA 555	0.97	0.31	-	0.57	-	0.37	-	0.63	-	0.37	-	0.63	-	-	-	-	-

Table S2 – Continued

Antibiotic	Isolate	>10 kDa					> 30 kDa				30 – 100 kDa				>100 kDa			
		MIC			FIC		MIC		FIC		MIC		FIC		MIC		FIC	
		**A**	**B**	**C**	**b**	**c**	**B**	**C**	**b**	**c**	**B**	**C**	**b**	**c**	**B**	**C**	**b**	**c**
	HA 1	250	9.37	-	0.29	-	9.37	-	0.29	-	11.6	-	0.30	-	4.68	-	0.27	-
	HA 501	5	0.19	1.87	0.29	0.50	0.46	2.5	0.34	0.63	0.46	-	0.34	-	0.15	2.5	0.28	0.63
	HA 549	12.5	0.93	1.87	0.32	0.27	0.62	2.5	0.30	0.33	0.46	-	0.29	-	0.23	2.5	0.27	0.33
Penicillin	HA 529	50	6.25	-	0.38	-	-	-	-	-	11.7	-	0.48	-	5.85	-	0.24	-
	CA 87	15.6	0.78	-	0.30	-	1.56	-	0.35	-	5.0	-	0.57	-	0.78	6.25	0.30	0.53
	CA 254	25	1.56	-	0.31	-	3.12	-	0.37	-	0.93	-	0.29	-	0.93	-	0.28	-
	CA 555	12.5	0.31	5	0.27	0.53	0.46	5	0.29	0.53	0.23	-	0.27	-	0.23	3.75	0.27	0.43

Table S3 - β-lactam-extract modulation, where A = initial antibiotic MIC; B = antibiotic MIC under modulation using 1/4 extract MIC; C = antibiotic MIC under modulation using 1/8 extract MIC, expressed as μg/mL . Both 'b' and 'c' FIC values correspond to the respective values seen in B and C, respectively (n=3).

Antibiotic	Isolate	>30 kDa PEE					30 – 100 kDa PEE				>100 kDa PEE			
		MIC			FIC		MIC		FIC		MIC		FIC	
		A	B	C	b	c	B	C	b	c	B	C	b	c
Amoxicillin	HA 1	250	9.37	37.5	0.39	0.28	9.37	-	0.29	-	1.17	37.5	0.25	0.28
	HA 501	5	0.5	1.87	0.45	0.50	0.75	1.87	0.40	0.50	0.25	1.25	0.30	0.38
	HA 549	7.8	0.37	1.87	0.40	0.36	0.5	1.87	0.31	0.36	0.37	1.25	0.30	0.29
	HA 529	25	11.7	-	0.82	-	5.86	-	0.48	-	1.95	-	0.33	-
	CA 87	25	0.75	9.37	0.38	0.50	0.75	9.37	0.28	0.5	0.75	9.37	0.28	0.50
	CA 254	25	0.37	9.37	0.36	0.50	0.75	9.37	0.28	0.5	0.37	-	0.26	-
	CA 555	3.13	0.37	-	0.47	-	0.37	-	0.37	-	0.18	-	0.31	-
Ampicillin	HA 1	250	9.37	37.5	0.39	0.28	-	-	-	-	1.17	37.5	0.25	0.28
	HA 501	5	0.5	1.25	0.45	0.38	0.75	1.87	0.40	0.50	0.25	0.93	0.30	0.31
	HA 549	3.9	0.37	0.93	0.44	0.36	0.5	1.25	0.38	0.44	0.25	1.25	0.31	0.45
	HA 529	25	7.81	-	0.66	-	5.85	-	0.48	-	1.95	-	0.33	-
	CA 87	15.6	0.75	9.37	0.40	0.73	0.75	12.5	0.30	0.92	-	9.37	-	0.73
	CA 254	15.6	0.75	9.37	0.40	0.73	0.75	9.37	0.30	0.72	0.25	-	0.27	-
	CA 555	3.9	0.37	1	0.44	0.38	0.37	-	0.34	-	0.18	1	0.30	0.38
Oxacillin	HA 1	62.5	2.93	31.2	0.40	0.62	11.7	31.25	0.44	0.62	0.29	23.4	0.25	0.50
	HA 501	0.97	0.5	-	0.87	-	0.5	-	0.77	-	0.37	-	0.63	-
	HA 549	0.63	0.37	-	0.94	-	-	-	-	-	0.37	-	0.84	-
	HA 529	50	23.4	-	0.82	-	11.7	-	0.48	-	3.90	-	0.33	-
	CA 87	12.5	1.87	-	0.50	-	1.87	-	0.40	-	1.25	-	0.35	-
	CA 254	15.6	1.25	-	0.43	-	1.87	-	0.37	-	1.25	-	0.33	-
	CA 555	0.97	0.37	-	0.73	-	0.37	-	0.63	-	0.18	-	0.44	-

Table S3 – Continued

Antibiotic	Isolate	>30 kDa PEE					30 – 100 kDa PEE				>100 kDa PEE			
		MIC			FIC		MIC		FIC		MIC		FIC	
		**A**	**B**	**C**	**b**	**c**	**B**	**C**	**b**	**c**	**B**	**C**	**b**	**c**
	HA 1	250	9.37	-	0.39	-	11.6	-	0.30	-	1.17	-	0.25	-
	HA 501	5	0.46	2.5	0.44	0.63	0.93	-	0.44	-	0.23	1.87	0.30	0.50
	HA 549	12.5	0.46	1.25	0.39	0.23	0.93	5	0.32	0.52	0.31	1.87	0.27	0.27
Penicillin	HA 529	50	-	-	-	-	-	-	-	-	9.37	-	0.44	-
	CA 87	15.6	3.12	6.25	0.55	0.53	4.68	6.25	0.55	0.52	1.56	6.25	0.35	0.53
	CA 254	25	9.37	9.37	0.72	0.50	9.37	-	0.62	-	1.17	12.5	0.30	0.63
	CA 555	12.5	0.62	5	0.40	0.53	0.62	3.75	0.30	0.42	0.31	5	0.27	0.53

Table S4 - β-lactam modulation, where A = initial antibiotic MIC; B = new antibiotic MIC using 1/4 extract; C = new antibiotic MIC using 1/8 extract, expressed as µg/mL. Both 'b' and 'c' FIC values correspond to the respective values seen in B and C, respectively (n=3).

		>30 kDa FEE					30 – 100 kDa FEE				>100 kDa FEE			
		MIC			FIC		MIC		FIC		MIC		FIC	
**Antibiotic**	**Isolate**	**A**	**B**	**C**	**b**	**c**	**B**	**C**	**b**	**c**	**B**	**C**	**b**	**c**
	HA 1	250	12.5	-	0.30	-	-	-	-	-	-	-	-	-
	HA 501	5	0.50	-	0.35	-	-	-	-	-	-	-	-	-
	HA 549	7.8	-	-	-	-	-	-	-	-	-	-	-	-
Amoxicillin	HA 529	25	11.7	-	0.72	-	-	-	-	-	-	-	-	-
	CA 87	25	-	-	-	-	-	-	-	-	-	-	-	-
	CA 254	25	-	-	-	-	-	-	-	-	-	-	-	-
	CA 555	3.13	-	-	-	-	-	-	-	-	0.25	-	0.33	-
	HA 1	250	12.5	-	0.30	-	-	-	-	-	-	-	-	-
	HA 501	5	0.50	-	0.35	-	-	-	-	-	-	-	-	-
	HA 549	3.9	-	-	-	-	-	-	-	-	-	-	-	-
Ampicillin	HA 529	25	-	-	-	-	-	-	-	-	-	-	-	-
	CA 87	15.6	-	-	-	-	-	-	-	-	-	-	-	-
	CA 254	15.6	-	-	-	-	-	-	-	-	-	-	-	-
	CA 555	3.9	-	-	-	-	-	-	-	-	0.37	-	0.34	-
	HA 1	62.5	-	-	-	-	-	-	-	-	-	-	-	-
	HA 501	0.97	-	-	-	-	-	-	-	-	-	-	-	-
	HA 549	0.63	-	-	-	-	-	-	-	-	-	-	-	-
Oxacillin	HA 529	50	23.4	-	0.72	-	-	-	-	-	-	-	-	-
	CA 87	12.5	3.75	-	0.55	-	-	-	-	-	-	-	-	-
	CA 254	15.6	3.75	-	0.49	-	-	-	-	-	-	-	-	-
	CA 555	0.97	-	-	-	-	-	-	-	-	0.37	-	0.63	-

Table S4 – Continued

Antibiotic	Isolate	>30 kDa FEE					30 – 100 kDa FEE				>100 kDa FEE			
		MIC			FIC		MIC		FIC		MIC		FIC	
		**A**	**B**	**C**	**b**	**c**	**B**	**C**	**b**	**c**	**B**	**C**	**b**	**c**
	HA 1	250	-	-	-	-	-	-	-	-	-	-	-	-
	HA 501	5	0.62	-	0.37	-	-	-	-	-	-	-	-	-
	HA 549	12.5	-	-	-	-	-	-	-	-	-	-	-	-
Penicillin	HA 529	50	-	-	-	-	-	-	-	-	-	-	-	-
	CA 87	15.6	4.68	-	0.55	-	-	-	-	-	1.56	-	0.35	-
	CA 254	25	4.68	-	0.43	-	-	-	-	-	-	-	-	-
	CA 555	12.5	-	-	-	-	-	-	-	-	0.62	-	0.30	-

Table S5. - Antibiotic modulation of extracts tested at 1/4 MIC against HA-MRSA isolates, where the respective MIC under modulation is listed as 'MICM', in addition to the fold-reduction (x-FR) from old antibiotic MIC (n=3). MIC and MICM concentrations expressed as µg/mL.

		HA 1			HA 501			HA 549			HA 529		
	**Extract (kDa)**	**MIC**	**MICM**	**x-FR**	**MIC**	**MICM**	**x-FR**	**MIC**	**MICM**	**x-FR**	**MIC**	**MICM**	**x-FR**
**Amoxicillin**	**Crude**		31.3	8.0		0.63	8.0		0.63	12.5		-	-
	**>10 CE**		62.5	4.0		0.31	16.0		0.72	10.8		9.37	2.7
	**30 – 100 CE**		3.90	64.0		0.75	6.7		0.38	20.8		7.80	3.2
	**30 – 100 PEE**	250	9.38	26.7	5	0.75	6.7	7.8	0.5	15.6	25	5.86	4.3
	**>30 CE**		4.69	53.3		0.38	13.3		0.38	20.8		5.86	4.3
	**>30 PEE**		9.37	26.7		0.5	10.0		0.38	20.8		11.7	2.1
	**>100 CE**		3.90	64.0		0.38	13.3		0.38	20.8		5.86	4.3
	**>100 PEE**		1.17	214.0		0.25	20.0		0.38	20.8		1.95	12.8
**Ampicillin**	**Crude**		31.3	8.0		1.56	3.2		0.31	12.5		6.25	4.0
	**>10 CE**		62.5	4.0		0.31	16.0		0.31	12.5		6.25	4.0
	**30 – 100 CE**		5.84	42.8		0.50	10.0		0.38	10.4		-	-
	**30 – 100 PEE**	250	9.37	26.7	5	0.75	6.7	3.9	0.50	7.8	25	5.85	4.3
	**>30 CE**		4.68	53.4		0.38	13.3		-	-		9.37	2.7
	**>30 PEE**		9.37	26.7		0.5	10.0		0.38	10.4		7.81	3.2
	**>100 CE**		4.68	53.4		0.38	13.3		0.38	10.4		-	-
	**>100 PEE**		1.17	214.0		0.25	20.0		0.25	15.6		1.95	12.8
**Oxacillin**	**Crude**		7.81	8.0		-	-		-	-		-	-
	**>10 CE**		1.95	32.0		0.31	3.1		0.15	4.0		15.6	3.2
	**30 – 100 CE**		1.95	32.0		0.13	7.8		-	-		23.4	2.1
	**30 – 100 PEE**	62.5	11.7	5.3	0.97	0.50	1.9	0.625	-	-	50	11.7	4.3
	**>30 CE**		2.93	21.3		0.50	1.9		-	-		11.7	4.3
	**>30 PEE**		2.93	21.3		0.50	1.9		0.38	1.7		23.4	2.1
	**>100 CE**		1.17	53.4		0.50	1.9		-	-		11.7	4.3
	**>100 PEE**		0.29	214.0		0.38	2.6		0.38	1.7		3.9	12.8

Table S5 Continued

		HA 1			HA 501			HA 549			HA 529		
	**Extract (kDa)**	**MIC**	**MICM**	**x-FR**	**MIC**	**MICM**	**x-FR**	**MIC**	**MICM**	**x-FR**	**MIC**	**MICM**	**x-FR**
**Penicillin**	**Crude**	250	125	2.0	5	0.19	25.6	12.5	0.19	64.0	50	25.0	2.0
	**>10 CE**		9.38	26.7		0.19	26.3		0.94	13.3		6.25	8.0
	**30 – 100 CE**		11.7	21.4		0.47	10.7		0.47	26.7		11.7	4.3
	**30 – 100 PEE**		11.7	21.4		0.94	5.3		0.94	13.3		-	-
	**>30 CE**		9.38	26.7		0.47	10.7		0.63	20.0		-	-
	**>30 PEE**		9.37	26.7		0.47	10.7		0.47	26.7		-	-
	**>100 CE**		4.68	53.4		0.15	32.0		0.23	53.4		5.85	8.5
	**>100 PEE**		1.17	214.0		0.23	21.3		0.31	40.0		9.38	5.3

Table S6. -Antibiotic modulation of extracts tested at 1/4 MIC against CA-MRSA isolates, where the respective new antibiotic MIC is listed, in addition to the fold-reduction (x-FR) from old antibiotic MIC (n=3). MIC and new MIC concentrations expressed as µg/mL.

		CA 87			CA 254			CA 555		
	**Extract (kDa)**	**MIC**	**MICM**	**x-FR**	**MIC**	**MICM**	**x-FR**	**MIC**	**MICM**	**x-FR**
**Amoxicillin**	**Crude**	25	3.13	8.0	25	3.13	8.0	3.13	-	-
	**>10 CE**		1.56	16.0		1.56	16.0		0.39	8.0
	**30 – 100 CE**		0.75	33.3		0.75	33.3		0.38	8.3
	**30 – 100 PEE**		0.75	33.3		0.75	33.3		0.38	8.3
	**>30 CE**		0.75	33.3		0.75	33.3		0.38	8.3
	**>30 PEE**		0.75	33.3		0.38	66.7		0.38	8.3
	**>100 CE**		1.17	21.4		0.5	50.0		0.38	8.3
	**>100 PEE**		0.75	33.3		0.38	66.7		0.19	16.7
**Ampicillin**	**Crude**	15.6	1.25	12.5	15.6	3.13	5.0	3.13	0.63	6.2
	**>10 CE**		1.56	10.0		1.56	10.0		0.39	10.0
	**30 – 100 CE**		0.75	20.8		0.75	20.8		0.38	10.4
	**30 – 100 PEE**		0.75	20.8		0.75	20.8		0.38	10.4
	**>30 CE**		0.38	41.7		0.38	41.7		0.25	15.6
	**>30 PEE**		0.75	20.8		0.75	20.8		0.38	10.4
	**>100 CE**		1.17	13.4		1.0	15.6		0.38	10.4
	**>100 PEE**		1.17	13.4		0.25	62.5		0.19	20.8
**Oxacillin**	**Crude**	12.5	-	-	15.6	5.0	3.1	0.97	-	-
	**>10 CE**		-	-		1.25	12.5		0.31	3.1
	**30 – 100 CE**		1.88	6.7		1.88	8.3		0.38	2.6
	**30 – 100 PEE**		1.88	6.7		1.88	8.3		0.38	2.6
	**>30 CE**		1.25	10		1.25	12.5		0.38	2.6
	**>30 PEE**		1.88	6.7		1.25	12.5		0.38	2.6
	**>100 CE**		2.5	5.0		1.88	8.3		-	-
	**>100 PEE**		1.25	10		1.25	12.5		0.19	5.2

Table S6 Continued

		CA 87			CA 254			CA 555		
	**Extract (kDa)**	**MIC**	**MICM**	**x-FR**	**MIC**	**MICM**	**x-FR**	**MIC**	**MICM**	**x-FR**
**Penicillin**	**Crude**	15.6	0.78	20.0	25	3.13	8.0	12.5	0.39	32.0
	**>10 CE**		0.78	20.0		1.56	16.0		0.31	40.0
	**30 – 100 CE**		5.0	3.1		0.94	26.7		0.23	54.3
	**30 – 100 PEE**		4.7	3.3		9.4	2.7		0.63	20
	**>30 CE**		1.56	10		3.13	8.0		0.46	27.2
	**>30 PEE**		3.13	5.0		9.37	2.7		0.63	20.0
	**>100 CE**		0.78	20.0		0.94	26.7		0.23	53.4
	**>100 PEE**		1.56	10		1.17	21.4		0.31	40.0

Table S7 - Additional genes exhibiting differentially expressed genes in the modulation complex compared to the control (p adj ≤ 0.05), which were not statistically significant (p adj ≤ 0.05) in any control comparisons.

Primary ID	ID Type	Gene/ Annotation	Feature type	Log2FC	p adj value	Proposed function
rpsP	Gene name	*rpsP*	CDS	-0.64	3.69×10^{-3}	Ribosomal dysfunction
rpsN	Gene name	*rpsN*	CDS	-0.84	2.52×10^{-2}	
clpP	Gene name	*clpP*	CDS	-0.91	1.24×10^{-2}	
rpmB	Gene name	*rpmB*	CDS	+0.68	1.83×10^{-2}	
rplS	Gene name	*rplS*	CDS	+0.66	2.25×10^{-2}	
rpmE	Gene name	*rpmE*	CDS	+0.63	4.72×10^{-2}	
holB	Gene name	*holB*	CDS	-0.60	1.28×10^{-2}	DNA replication inhibition
rpmG	Gene name	*rpmG*	CDS	-0.60	3.46×10^{-2}	
norB	Gene name	*norB*	CDS	-0.90	1.99×10^{-3}	
adcA	Gene name	*adcA*	CDS	-0.46	3.70×10^{-2}	Active transport
cntA	Gene name	*cntA*	CDS	-0.42	4.80×10^{-2}	
cydC	Gene name	*cydC*	CDS	+0.57	5.00×10^{-2}	
cydD	Gene name	*cydD*	CDS	+0.85	1.20×10^{-2}	
fetB	Gene name	*fetB*	CDS	+0.87	4.80×10^{-2}	
nikA	Gene name	*nikA*	CDS	-0.65	5.00×10^{-2}	
opp3b	Gene name	*opp3b*	CDS	-0.52	1.00×10^{-2}	
patR	Gene name	*patR*	CDS	-0.50	3.40×10^{-2}	
tagH	Gene name	*tagH*	CDS	-0.76	1.20×10^{-2}	
yhaP	Gene name	*yhaP*	CDS	-0.50	3.50×10^{-2}	
yhgE	Gene name	*yhgE*	CDS	+0.79	3.50×10^{-2}	
